# Large-Scale Plasma Proteome Epitome Profiling is an Efficient Tool for the Discovery of Cancer Biomarkers

**DOI:** 10.1016/j.mcpro.2023.100580

**Published:** 2023-05-20

**Authors:** Jozsef Lazar, Peter Antal-Szalmas, Istvan Kurucz, Annamaria Ferenczi, Mihaly Jozsi, Ilona Tornyi, Monika Muller, Janos Tibor Fekete, John Lamont, Peter FitzGerald, Anna Gall-Debreceni, Janos Kadas, Andras Vida, Nadege Tardieu, Yann Kieffer, Anne Jullien, Mariana Guergova-Kuras, William Hempel, Andras Kovacs, Tamas Kardos, Nora Bittner, Eszter Csanky, Maria Szilasi, Gyorgy Losonczy, Klara Szondy, Gabriella Galffy, Edit Csada, Klara Szalontai, Attila Somfay, David Malka, Paul Cottu, Krisztina Bogos, Laszlo Takacs

**Affiliations:** 1 Biosystems International Kft., Debrecen, Hungary; 2 Biosystems Immunolab Zrt., Debrecen, Hungary; 3 Department of Laboratory Medicine, Faculty of Medicine, University of Debrecen, Debrecen, Hungary; 4 Department of Immunology, ELTE Eötvös Loránd University, Budapest, Hungary; 5 MTA-ELTE Complement Research Group, Eötvös Loránd Research Network (ELKH), Budapest, Hungary; 6 Department of Human Genetics, Faculty of Medicine, University of Debrecen, Debrecen, Hungary; 7 Adware Research Kft., Balatonfüred, Hungary; 8 Department of Bioinformatics, Semmelweis University, Budapest, Hungary; 9 Randox Laboratories Ltd, Crumlin, United Kingdom; 10 Biosystems International SAS, Evry, France; 11 Department of Pulmonology, Faculty of Medicine, University of Debrecen, Debrecen, Hungary; 12 Department of Pulmonology, Miskolc Semmelweis Hospital and University Hospital, Miskolc, Hungary; 13 Department of Pulmonology, Faculty of Medicine, Semmelweis University, Budapest, Hungary; 14 Csongrád County Hospital of Chest Diseases, Deszk, Hungary; 15 Department of Pulmonology, Faculty of Medicine, University of Szeged, Deszk, Hungary; 16 Department of Medical Oncology, Gustave Roussy, Villejuif, France; 17 Department of Medical Oncology, Institut Curie, Paris, France; 18 National Koranyi Institute for Pulmonology, Budapest, Hungary

**Keywords:** proteoform, protein variants, epitope, plasma epitom profiling, cancer, lung cancer, biomarker

## Abstract

Current proteomic technologies focus on the quantification of protein levels, while little effort is dedicated to the development of system approaches to simultaneously monitor proteome variability and abundance. Protein variants may display different immunogenic epitopes detectable by monoclonal antibodies. Epitope variability results from alternative splicing, posttranslational modifications, processing, degradation, and complex formation and possesses dynamically changing availability of interacting surface structures that frequently serve as reachable epitopes and often carry different functions. Thus, it is highly likely that the presence of some of the accessible epitopes correlates with function under physiological and pathological conditions. To enable the exploration of the impact of protein variation on the immunogenic epitome first, here, we present a robust and analytically validated PEP technology for characterizing immunogenic epitopes of the plasma. To this end, we prepared mAb libraries directed against the normalized human plasma proteome as a complex natural immunogen. Antibody producing hybridomas were selected and cloned. Monoclonal antibodies react with single epitopes, thus profiling with the libraries is expected to profile many epitopes which we define by the mimotopes, as we present here. Screening blood plasma samples from control subjects (n = 558) and cancer patients (n = 598) for merely 69 native epitopes displayed by 20 abundant plasma proteins resulted in distinct cancer-specific epitope panels that showed high accuracy (AUC 0.826–0.966) and specificity for lung, breast, and colon cancer. Deeper profiling (≈290 epitopes of approximately 100 proteins) showed unexpected granularity of the epitope-level expression data and detected neutral and lung cancer–associated epitopes of individual proteins. Biomarker epitope panels selected from a pool of 21 epitopes of 12 proteins were validated in independent clinical cohorts. The results demonstrate the value of PEP as a rich and thus far unexplored source of protein biomarkers with diagnostic potential.

## Highlights

• Available surfaces represent epitope variability accessible to mAb-s.
• Epitopes of abundant plasma proteins carry biomarker value.
• Epitope-defined variability analysis increases the resolution of proteome profiling.
• Approachable epitopes have a surprising potential as diagnostics.
• Antibody libraries specific to epitomes provide a fine tool to screen for biomarkers.

## Graphical Abstract

**Figure:**
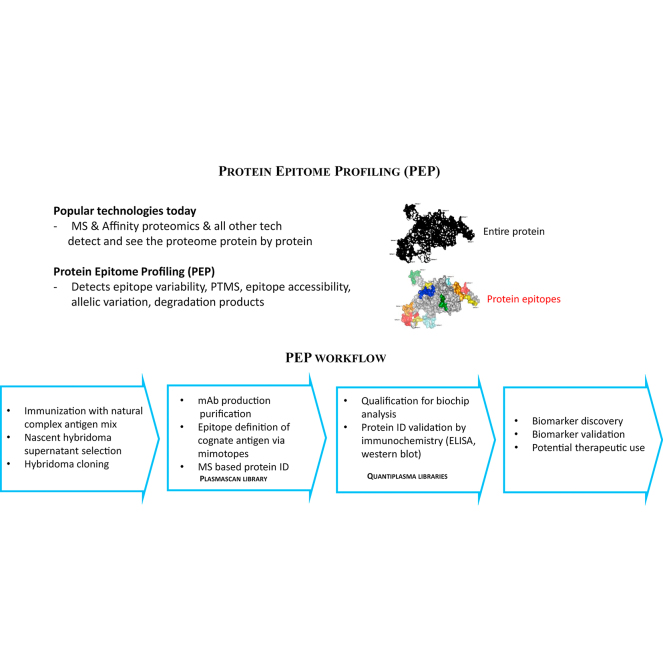

## In Brief

Mass spectrometry–driven proteomics today focuses on the quantification of protein levels, while little effort is dedicated to the development of system approaches to simultaneously monitor proteome variability and abundance on the global scale. Here, we present a robust and analytically validated protein epitome profiling technology (PEP). We show that PEP detects immunogenic epitope variability, provides increased resolution for proteome analysis, and represents a rich source for cancer-specific biomarker discovery, delivering binders with apparent ease of translatability.

Global genome and transcriptome profiling by next-generation sequencing reached the bedside in the form of approved tests with proven clinical utility (1, 2, 3, 4, 5, 6). As proteins are the most frequent effectors of biological function, it is expected that proteome profiling will provide important clinical value for assessing actual disease status. However, the task remains challenging; the translation of global proteomic data is inefficient. There are approximately 20,000 protein-coding genes in humans, but the number of actual proteins is estimated to be 100- to 1000-fold greater due to protein variants displaying remarkable epitope variability (7, 8). Epitopes are the molecular structures within an antigen that make specific contacts with the antibodies and may interact with functional partners (9), The source of epitope variability similarly to that of proteoforms is comprised of (i) “*in trans*” genetically coded variations, such as alternative splicing, different translational start and stop points, allelic protein species, and (ii) “*cis*-coded” and stochastically influenced alternative forms, including posttranslational modifications, variable multi-chain association, regulated processing, degradation (10), and, in addition, three-dimensional conformation changes and protein–protein complex formation (11). Important to note that while proteoforms are distinct protein species, epitopes represent sub-protein structures. Moreover, current proteomic technologies focus on the global detection of quantifiable representational differences (12), and they neglect the impact of epitope and protein variability where functional and clinical relevance is often hidden. The importance of global proteome variability testing is already and clearly illustrated by the establishment of the Human Proteoform Project (13). Presently, the most popular technologies are based on mass spectrometry (MS). Although MS-based approaches are global and hypothesis-free and provide an estimate of protein levels, neither MS technologies (top-down or bottom-up) nor current affinity reagent-based methods, such as recombinant phage (14, 15), recombinant antibody (16, 17, 18, 19), and SomaScan (20, 21, 22, 23) technologies, address and detect natural immunogenic epitope variability on a global scale.

All forms of protein variation affect epitopes by inducing three-dimensional structural changes and the modification of topologic accessibility (24, 25, 26). Protein epitome profiling (PEP) is the first effort to profile the epitome with monoclonal antibody (mAb) libraries for the discovery of functionally and clinically relevant epitopes and to detect variations in the antigenic epitome associated with cancer for the further development of accurate and individual cancer-specific diagnostic biomarker panels. To achieve PEP, we used normalized human plasma as a complex immunogen. Resulting hybridoma supernatants were selected for mAb production and the corresponding hybridomas were cloned. Monoclonal antibodies react with single epitopes, thus profiling with the libraries is expected to profile many epitopes which we define by the mimotopes, as we present here. Monoclonal antibodies produced by cloned hybridomas were then qualified for biochip use, which we deployed for profiling the epitome of individual plasma samples from control subjects and cancer patients. The graphical abstract describes the PEP process.

## Experimental Procedures

### Cohorts and Biobanks

#### NKTH Cohort

Biobanked blood samples were collected along with informed consent approved by the Regional and Institutional Ethics Committee of the Medical and Health Science Center, University of Debrecen (DE OEC RKEB/IKEB, permit No: 3049-2009, 3140-2010), Hungary, the study abided by the WMA Declaration of Helsinki principles. The biobanks contained samples from 153 LC, 107 colon cancer, and 106 breast cancer patients. Patients were enrolled following cancer diagnosis by histopathological (biopsy) and imaging criteria. Standard EDTA (BD Vacutainer Plastic K_2_EDTA Tube, 10 ml) plasma samples were collected at the time of enrollment, before starting treatment, and stored at −80 °C until use. In total, 500 controls were enrolled (330 healthy individuals and 119 individuals with diabetes mellitus); the 330 healthy individuals served as controls for the patients with cancer in the study reported here. Strict inclusion criteria were applied for the healthy controls; only individuals without any apparent diseases (physical signs and anamnestic history of having no cancer) were included. Patients were matched with respect to age and sex with the control subjects.

#### BioDiagnostica Cohort

The cohort was assembled from four centers (Semmelweis Hospital, Miskolc, Hungary and the Departments of Pulmonology of the University of Debrecen, University of Szeged, and Semmelweis University, Budapest, Hungary), the permit was approved by the Medical Research Council of Hungary (ETT TUKEB, permit No: 11739/2014/EKU, 107/2014 and 417/2014), and the study abided by the WMA Declaration of Helsinki principles. The biobank included 908 standard EDTA (BD Vacutainer Plastic K_2_EDTA Tube, 10 ml) samples: 425 LC and 483 controls. The blood samples were collected following the diagnosis of LC by histological, molecular, and imaging criteria, but before the start of treatment. All participants had given their informed consent. In this project, controls with or without COPD were enrolled, making it possible to assess the impact of COPD, as patients with LC frequently have COPD. Sub-cohorts: in addition to age and sex, the sub-cohorts were matched for BMI and smoking habit with respect to the COPD stage. The guidance of the American Joint Committee on Cancer was used for LC staging (27).

### Hybridoma and QuantiPlasma Libraries

Mouse monoclonal antibody-producing hybridomas were generated with standard, but still recently reviewed procedures (28, 29).

Specifically, the production of BSI’s hybridoma library was described previously (30, 31, 32). Members of the QuantiPlasma libraries, QP69 & QP300, were selected based on their performance in a high-throughput automated 384-well microplate-based CIA (HTS-CIA) with total plasma tracer or depleted plasma tracers. The HTS-CIA protocol was modified from the HTS ELISA described previously (30). Briefly, 384-well high protein-binding plates (Corning Inc) were coated with goat anti-mouse IgG γ-chain specific polyclonal antibody (goat anti-mouse IgG) (Southern Biotechnology Associates, Inc) for 2 h at room temperature. Following washing, the wells were blocked with 0.5% BSA in PBS at 4 °C overnight. Undiluted culture supernatants or purified mAb IgG (1 μg/ml in PBS) were added in quadruplicate. Mouse anti-human mAb against albumin (Zymed Laboratory Inc) was added to eight wells on each plate and used as a positive control. A tissue culture medium was also added to eight wells as a negative control. The plates were incubated overnight at 4 °C and, following washing, all wells were incubated with tracers (biotinylated depleted plasma) mixed with dilutions (10× and 1000×) of normal human plasma. Biotinylated human serum albumin was added to the wells containing the positive controls and the incubation continued for 90 min at room temperature. Unbound protein was then removed by washing and horseradish peroxidase–coupled avidin (Vectastain Elite ABC peroxidase kit from Vector Laboratories) was added to each well for the detection of bound biotinylated proteins as specified by the vendor’s protocol. Following washing, color development was carried out by adding freshly prepared substrate solution (H_2_O_2_; Sigma) and chromogen (*o*-phenylenediamine, Sigma) to each well. The reaction was followed by kinetic reading for 4 min at 450 nm at 37 °C, at 32-s intervals, using ELISA readers from Molecular Devices (Toronto, Canada). All amenable steps were automated. The V_max_ was calculated from the linear portion of the curves using SoftMax Pro software from Molecular Devices. V_max_ values were used as input for the four-parameter logistic (4PL) Hill-Slope model to determine the linearity and quality of the inhibition. Qualifying mAbs that exhibited linear inhibitory curves (R^2^ > 0.9) in the sbCIA with total or depleted tracers were selected as members of the QP69 or QP300 libraries, respectively.

### Mouse mAb Purification

Selected cloned hybridoma cell lines were used for either *in vivo* mAb production from mouse ascites as it was described previously (30) or *in vitro* from high IgG-containing supernatants harvested from BD Falcon CELLine flask. Almost all mouse monoclonals were of IgG1 isotype. Purification steps of IgG from the supernatant were started from the second step of the affinity chromatography purification procedure referenced previously. Experimental procedures involving laboratory mice had been reviewed and accepted by the University of Debrecen Committee of Animal Welfare (3/2006/DE MÁB, 15/2011/DE MÁB, 3-1/2017/DEMÁB).

### Tracer Preparation

For the preparation of tracers, pooled plasma was used from 50 blood donors. For the total plasma tracer, 10 mg of plasma protein was biotin labeled with EZ-link Sulfo-NHS-Biotin (Thermo Scientific) according to the manufacturer’s recommendations. After unbound biotin removal by Zeba Spin Desalting Columns (Thermo Scientific), the biotinylated proteins were concentrated using an Amicon Ultracel 3K (Millipore UFC800324) centrifugal filter unit, aliquoted, and stored at or below −70 °C. For the depleted tracer preparation, the 14 most abundant plasma proteins were first removed from the pooled plasma using a Human 14 Multiple Affinity Removal System Column (Agilent Technologies, 5188-6559) according to the manufacturer’s protocol. Flowthrough fractions were pooled and the buffer was switched to PBS (SnakeSkin 10,000 MWCO, Thermo Scientific). Two-milligram portions of the depleted protein were then labeled with biotin and stored as already described.

### Cognate Protein Identification

Antigen identification was performed by immune affinity purification of the cognate antigen of the mAbs, as described earlier (31), followed by mass spectrometry analysis of the eluted protein(s) on the following LC-MS/MS instruments.

#### Agilent 1000 LC + 4000QTRAP Mass Spectrometer

The lyophilized trypsin-digested samples were reconstituted in 10 μl Solvent A (LC water containing 0.1% formic acid). 5 μl of the reconstituted sample was mixed with 5 μl Solvent A and 8 μl mix was injected for LC-MS/MS analysis.

Before mass spectrometric analysis, peptides were separated using a 100-min water/acetonitrile gradient. Peptides were first desalted and concentrated on a reverse phase C18 column (5 × 0.3 mm, 5 μm particle size, Agilent), then separated on Zorbax 300SB-C18 (150 mm × 75 μm 3.5 μm particle size, Agilent). The chromatographic separation was performed using a gradient of 0 to 30% solvent B over 65 min, followed by a rise to 100% of solvent B over 5 min, followed by a 5 min rise to 100% of solvent B. After which the system returned to 100% solvent A in 5 min for a 20 min hold-on. Solvent A was 0.1% formic acid in LC water and solvent B was LC acetonitrile containing 0.1% formic acid. The flow rate was set to 0,3 μl/min.

Positive mode LC-MS/MS scans were performed on a 4000 QTRAP (ABSciex) mass spectrometer using a NanoSpray II MicroIon source, controlled by the Analyst 1.4.2 software (ABSciex). The spray voltage was 2500 V, the sheath gas was 40 psi, the curtain gas was 10 psi and the source temperature was 150 °C. Information Dependent Acquisition method was utilized; after the first mass scan (mass range 440–1400 m/z), an enhanced resolution experiment was carried out to establish the charge state of the precursor ions. The MS/MS spectra of the five most intensive ions were recorded (mass range 100–2000 amu) in Enhanced Product Ion mode at a scan rate of 4000 amu/s with 30 eV collision energy.

MS/MS spectra were searched against NCBInr 20090314 (8016074 sequences; 2759887765 residues) using a web-based Mascot Server (version 2.4, Matrix Science). One missed cleavage was allowed, Carboxymethyl (C) modification was set as fixed modification, N-terminal acetyl, Gln->pyro-Glu (N-term Q), and oxidation (M) as variable modifications. Mass tolerances used for precursor ions ±1.2 Da and fragment ions ±0.6 Da.

Peptides were manually identified based on the b and y ion series and at least 2 peptides were required to be identified to accept that the protein was present in the sample.

#### nanoAcquity UPLC - LCQ-Fleet MS

For in-gel digestion, the protein bands of interest were excised. The proteins were reduced by incubation with 10 mM DTT for 30 min at 56 °C and then alkylated with 55 mM iodoacetamide (IAA) for 30 min at room temperature in the dark. Trypsin was added to the gel pieces and after 15 min incubation at 4 °C, the digestion proceeded for 4 h at 37 °C. Tryptic peptides were extracted with 2% formic acid in 50% acetonitrile with shaking. The extracts were completely dried using a Speed Vac and redissolved in 0.1% formic acid for LC-MS/MS analysis.

For LC-MS/MS analysis, an LCQ-Fleet mass spectrometer (Thermo Fisher Scientific) online coupled with a nanoAcquity (Waters) UPLC system was used. The samples were analyzed using a data-dependent triple play method: after each survey scan the 3 most intense peaks were selected for zoom scan and for CID fragmentation using 35% normalized collision energy. Dynamic exclusion was utilized with an exclusion duration of 30 s and 2 repeat counts.

The raw files were converted to mgf peak list files using Mascot Distiller (ver.: 2.2.1.0) which were subjected to database search on our in-house Mascot (ver.:2.2.04) server using the NCBI human database (NCBInr 100,220, 183,553 sequences were searched). The following search parameters were set: enzyme: Trypsin with a maximum of 2 missed cleavage sites; Carbamidomethylation was set as a constant modification of Cys residues, and several variable modifications were set: acetyl (Protein N-term), Gln->pyro-Glu (N-term Q), oxidation (M). Mass tolerances were 0.6 Da and 1 Da for the peptides and fragment masses respectively. Protein scores are derived from ions scores as a non-probabilistic basis for ranking protein hits. The significance threshold was set to 5% (*p* < 0.05)

#### nanoAcquity UPLC - QTOF Permier

Before the nano LC-MS(MS) measurements 10 μl IPP eluates were in-solution digested. Samples were unfolded and reduced with 3 μl 0.5% RapiGest SF and 1.5 μl 100 mM 1,4-dithio-*LD*-threitol (DTT) at 60 °C for 30 min. In the next step, alkylation was performed by adding 4 μl 200 mM NH_4_HCO_3_ and 2 μl 200 mM 2-iodoacetamide (IAA) at room temperature for 30 min in the dark. Then the alkylated samples were digested by trypsin (2 μl, 40 μM) or by chymotrypsin (2 μl, 39 μM) at 37 °C for 180 min. The digestion was quenched by adding 1 μl formic acid (30 min at 37 °C). The reaction product was centrifuged at 13,500 rpm (corresponding to 17,000*g*) for 10 min.

The digested peptide mixtures were analyzed using a nanoflow UHPLC system (nanoAcquity UPLC, Waters) coupled with a high-resolution QTOF Premier mass spectrometer (Waters). The electrospray emitter was purchased from New Objective, Woburn, USA. First, the 1 μl injected peptide mixtures were desalted online on a Symmetry C18 trap column (180 μm i.d. × 20 mm, Waters). Then the peptides were separated on a reverse phase analytical column (C18, 75 μm i.d. × 200 mm, 1.7 μm BEH particles, Waters) using a flow rate of 450 nl/min and column temperature of 55 °C. The applied gradient was the following: first, a 5 min long gradient going from 0% to 10% solvent B, followed by a 65 min long gradient going from 10% to 50% solvent B. This was followed by washing and equilibration steps (solvent B increased to 85% in 2 min, kept there for 18 min, and finally returned to 0% B in 2 min, kept there for 18 min). Solvent A was water containing 0.1% formic acid and solvent B was acetonitrile also containing 0.1% formic acid.

The mass spectrometer operated in positive electrospray ionization mode. Peptides were measured performing both single-stage mass spectrometry in extended dynamic range mode and tandem mass spectrometry. The capillary voltage was 2.8 kV, nanoflow 0.3 bar, source temperature: 90 °C. Sequences of peptides were determined using tandem mass spectrometry in the so-called survey mode (DDA survey). The parent ion was selected in the *m/z* 400 to 2000 range, and MS/MS spectra were acquired in the *m/z* 150 to 2000 range. The collision gas was argon, at 4∗10^−3^ mbar.

MS/MS survey data were processed using ProteinLynx Global Server v.2.3 (Waters) and searched against version 2011_01 of the SwissProt sequence database with human taxonomy using Mascot Server version 2.2 (Matrix Science). One missed cleavage was allowed, carbamidomethyl (C) modification was set as fixed modification, and oxidation (M) as variable modification. Mass tolerances for precursor ions and fragment ions are 7 ppm and 0.1 Da respectively. The protein false discovery rate was set to be 1% (peptide decoy was on), and the peptide significance threshold was set to *p* < 0.05.

#### Dionex Ultimate 3000 nanoRSLC - Bruker Maxis II ETD

In-solution digestion was carried out using 10 μl IPP eluates. Samples were unfolded and reduced with 0.7 μl 0.5% RapiGest SF and 0.5 μl 100 mM 1,4-dithio-*LD*-threitol (DTT) at 60 °C for 30 min. In the next step, alkylation was performed by adding 3.9 μl 200 mM NH_4_HCO_3_ and 1.1 μl 200 mM 2-iodoacetamide (IAA) at room temperature for 30 min in the dark. Then, the alkylated samples were digested by LysC-Trypsin mixture (0.5 μl, 50 ng/ml) at 37 °C for 60 min and then by trypsin (0.5 μl 200 ng/ml) at 37 °C for 120 min. The digestion was quenched by adding 1 μl formic acid (30 min at 37 °C). The reaction product was centrifuged at 13,500 rpm (corresponding to 17,000*g*) for 15 min.

The digested peptide mixtures were analyzed using Dionex Ultimate 3000 nanoRSLC (Dionex) coupled to a Bruker Maxis II ETD mass spectrometer (Bruker Daltonics GmbH) *via* CaptiveSpray nano booster ion source. First, the 1 μl injected peptide mixtures were desalted online on a Symmetry C18 trap column (180 μm i.d. x 20 mm, Waters). The 1 μl peptide mix was separated on an ACQUITY UPLC M-Class Peptide BEH C18 Column (130 Å, 1.7 μm, 75 μm X 250 mm, Waters) using a flow rate of 300 nl/min and column temperature of 48 °C. The applied gradient was the following: first, a 11-min long flow going at 4% solvent B, followed by a 90 min long gradient going from 4% to 50% solvent B. This was followed by washing and equilibration steps (solvent B increased to 90% in 1 min, kept there for 5 min, and finally returned to 4% B in 1 min, kept there for 20 min). Solvent A was water containing 0.1% formic acid and solvent B was acetonitrile also containing 0.1% formic acid.

The mass spectrometer operated in positive electrospray ionization mode. Peptides were measured performing both single-stage mass spectrometry in extended dynamic range mode and in tandem mass spectrometry. The capillary voltage was 1.3 kV, nanoflow 0.0 bar, source temperature: 150 °C. Sequences of peptides were determined using tandem mass spectrometry in data-dependent analysis mode, which was performed using 2.5 s cycle time. The MS spectra scan speed was set to 3.00 Hz, and for the MS/MS spectra, it was set to 4.00 Hz for low-intensity peaks (7500–40,000 cts) or 16.00 Hz for high-intensity peaks (above 40,000 cts).

MS/MS survey data was processed using ProteinScape 4.0 (Waters) and searched against version 20.01.2016 of SwissProt sequence database with human taxonomy using Mascot Sever version 2.5 (Matrix Science). Two missed cleavages were allowed, carbamidomethyl (C) modification was set as fixed modification, deamidation (N, Q), and oxidation (M) as variable modifications. Mass tolerances for precursor ions and fragment ions are 7 ppm and 0.05 Da respectively. The protein false discovery rate was set to be 1% (peptide decoy was on), and the peptide significance threshold was set to *p* < 0.05.

### Epitope/Mimotope Identification and Frequency Calculation

The PhD-12 Phage Display Peptide Library Kit (New England Biolabs) was used to identify mimotope peptide sequence(s), as previously reported in detail (33). For each mAb at least 12 phage clones were sequenced. The obtained 12-mer peptide sequences of each screened mAbs were first ranked and then the unique sequence(s) or motif(s) were determined. Unique sequence(s) of each antibody were used to determine epitope-level redundancy based on the frequencies of common mimotope peptide(s) of compared mAb pairs according to the following formula:

RedXY=[(Xn1∗Ym1)∗100]+...+[(Xni∗Ymi)∗100]

where

Xn = the frequency of the nth peptide of mAb X (number of unique mimotope peptides n divided by the number of all peptides of mAb X)

Ym = frequency of the mth peptide of mAb Y (number of unique mimotope peptides m divided by the number of all peptides of mAb Y)

Xn1 = the frequency of the first common mimotope peptide of mAb X between mAbs X & Y

Ym1 = the frequency of the first common mimotope peptide of mAb Y between mAbs X & Y

Xni = the frequency of the ith common mimotope peptide of mAb X between mAbs X & Y, and

Ymi = the frequency of the ith common mimotope peptide of mAb Y between mAbs X & Y.

A list of the unique peptides of the QP library mAbs can be found in supplemental Table S1. A database containing all peptide data shown in supplemental Fig. S2. can be found there.

### Surface Plasmon Resonance Experiments

#### Peptide Immobilization on the Chip Surface

BiaCore precoated streptavidin chips (BiaCore SA) were used for the immobilization of peptides. Before immobilization, the surface was pretreated with a 1 M NaCl, 50 mM NaOH solution to remove any contamination. For immobilization, 10 μl of a 10 μg/ml biotinylated peptide solution (BioTide, JPT Peptide Technologies GmbH) was used to reach saturation on the flow-cell surface. The peptides were immobilized on the surface of the flow cell 1 (Fc1), while flow cell 2 (the reference cell, Fc2) remained intact. If the amount of immobilized peptide decreased, the immobilization procedure was repeated.

#### Determination of Binding Curves Using Various Dilutions of the Antibodies

Binding experiments were performed in HBS-EP buffer (10 mM HEPES, 0.15 M NaCl, 3 mM EDTA, 0.005% Surfactant P20 (= Tween 20), pH 7.4). The flow rate was 10 μl/min for both the association and dissociation phases during analysis of the antibody-peptide interaction. The antibody preparations were diluted to 666, 266, 133, 66, 27, and 13 nM in HBS-EP (4 or 5 dilutions for each pair). Calculation of the dissociation constants was performed from data collected for 120 s after the termination of ligand injection.

#### Chip Regeneration and Storage

The sensor chip surface was regenerated using 50 mM NaOH, which was injected onto the chip at a flow rate of 10 μl/min for 1 min.

Sensor chips were stored under nitrogen atmosphere at 4 ^°^C according to the manufacturer’s instructions. Using this storage method, the chips with immobilized peptides can be used for approximately 1 month based on our experience.

#### Determination of the K_D_ Values

K_D_ values were determined using the BIA evaluation program. The “two-state reaction (conformation change)” model was applied for the peptide-antibody interaction.

#### Plasma Competition Assays

Plasma competition assays were run at various antibody concentrations (266, 133, and 27 nM) while adding various amounts of plasma in the range of 0 to 20%. The competition values were calculated from the decrease in the resonance signal at the beginning of the dissociation phase. The signals were plotted against the plasma concentration and the IC_50_ values were determined using a sigmoidal fit.

#### Protein Competition Assays

Protein competition assays were performed similar to the plasma competition assays. The antibody concentrations were 133 and 27 nM for complement factor H competition. The competing protein: antibody ratio varied between 1:40 and 5:1. IC_50_ values were calculated analogous to the plasma competition assays. All binding pairs were also tested by plasma and protein competition.

### Binding of BSI Monoclonals to CFH, CFH Domains, and Different FHR Proteins

Human factor H (FH) was purchased from Merck (Budapest, Hungary). Recombinant human FHR-5 was purchased from R&D Systems (Biomedica; Budapest, Hungary). Recombinant FH fragments covering the complement control protein (CCP) domains 1 to 4 (FH1-4) and 15 to 20 (FH15–20), recombinant FHR-1, and FHR-4B were generated and produced in insect cells and purified by nickel-affinity chromatography, as described previously (34, 35, 36, 37).

#### ELISA

To identify binding sites and cross-reactivity of the factor H (FH)-specific mAbs, human serum albumin was used as a negative control protein, and human FH, FH CCP1-4, FH CCP15 to 20, FHR-1, FHR-4B, and FHR-5 were immobilized at 4 μg/ml on Nunc MaxiSorp plates (Thermo Fisher Scientific). Monoclonal anti-FH antibodies were added at 1 μg/ml and their binding was detected with HRP-conjugated goat-anti-mouse IgG antibody (Dako) by the addition of TMB PLUS substrate (Kem-En-Tec Diagnostics). Absorbance was measured at 450 nm using a Thermo Multiskan EX microplate reader (Thermo Fisher Scientific).

### CFH Concentration Measurement

#### Commercial ELISA

The CFH concentration of selected samples was measured using a commercially available complement factor H, human, ELISA kit (Hycult Biotech), following the manufacturer’s protocol.

#### BSI ELISA

Plasma sample CFH concentrations were determined in sandwich ELISA assays employing BSI mAbs. For CFH protein capture 10 μg/ml Bsi0885, Bsi0862, or Bsi0397 was coated. Plasma samples were tested at 20,000-fold dilutions, along with the Hycult kit’s CFH calibrator protein. Bsi0885 and Bsi0862 captured CFH was detected by biotinylated Bsi0747 at 2 μg/ml, and Bsi0397 captured CFH by biotinylated Bsi1328 at 5 μg/ml with HRP conjugated streptavidin (Thermo Fisher Scientific) utilizing TMB solution from TMB tablets (Sigma). The colorimetric reaction was stopped with 4N sulfuric acid solution then absorbance was detected by Thermo Multiskan Ascent Microplate Reader at 450 nm.

### Experimental Design and Statistical Rationale

For cognate protein identification, QPLC21 mAbs were used to pull down cognate proteins from human plasma. The IP eluates then were either used directly or as gel slices cut out from Commassie or Silver stained SDS PAGE gel to analyze on different LC-MS/MS setups as it was described in “Cognate protein identification” section.

Technical replicate of cognate protein ID determination was done in the case of four mAbs on LC-MS/MS instruments obtaining similar protein hit (Bsi0097, Bsi0190, Bsi0300, Bsi0789). Importantly, MS analysis-derived cognate protein binding was verified in ELISA experiments as biological replicates, employing commercially available purified human plasma protein directly coated to the surface of microwells (supplemental Table S4).

Lung and other major cancer-type recognition of QuantiPlasma monoclonal antibody library members, printed on QP69 and QP300 biochips, were determined in the CIA assay of the NKTH sample cohort. Twenty-one mAb sets (QPLC21), which discriminate between LC and healthy control individuals, were further characterized with BD cohort samples. Each sample was measured three times, and replicate RLU/RLU_max_ % values were averaged and used for statistical evaluation of mAbs individually as well as included in composite models.

### QuantiPlasma Biochip Experiments

QP69 and QP300 biochips array kits using BSI’s QuantiPlasma monoclonal antibody library were produced by Randox Ltd, as described previously (38). QP69 mAbs were spotted on three biochip arrays, while QP300 mAbs were spotted on 18 biochip arrays due to the limited number of testing regions on a 9 × 9 mm biochip surface. For each 18-sample measurement position (6 biochip racks handled at a time: 6 × 9 chips = 54 chips/3 chips of the QP69 mAbs = 18 samples), two positions were used to measure maximal signal intensities for quality control and normalization purposes for QP69 biochip measurements. For QP300, six biochip racks provided only three measurement positions (6 × 9 chips = 54 chips/18 chips of the QP300 mAbs = 3 samples); therefore, one position was used for maximal signal intensity and two for sample measurements. The biochips were processed according to the supplied protocol. Briefly, the biotin-labeled tracer was reconstituted with 1 ml of deionized water and incubated for 30 min at room temperature on an orbital shaker. Six biochip racks (nine biochips assembled together with a handle) were inserted into the handling tray and 200 μl assay diluent buffer (19 mM Tris-buffered saline pH 8 containing a protein matrix, surfactant, and preservatives), 50 μl diluted sample, and 50 μl reconstituted tracer were added to each biochip. Samples were diluted 100- and 1000-times for the QP69 assay and 300-times for the QP300 assay. After a 1-h incubation in a Randox biochip thermoshaker at 37 °C and 370 RPM, the solution was discarded and the biochips were quickly washed six times with approximately 350 μl washing buffer/biochip well (20 mM Tris-buffered saline pH 7.2, containing surfactant and preservatives) followed by six rinses for 2 min with approximately 350 μl washing buffer/biochip well by gentle tapping of the edges of the handling tray for 10 to 15 s and then leaving them to soak. After the last washing cycle, the residual wash buffer was removed by tapping the handling tray with the biochip racks upside down onto lint-free tissue paper. Then, the biochips were incubated with 300 μl conjugate buffer (19 mM Tris-buffered saline pH 7.2, containing a protein matrix, surfactant, preservatives, and assay-specific reagents labeled with horseradish peroxidase) for 1 h in a Randox thermoshaker at 37 °C and 370 RPM. Then, the previously described washing steps were repeated. Wash buffer (350 μl) was then added to avoid the drying of the chip surface. Imaging was performed within 30 min after the last washing step by incubating one biochip rack of biochips at a time with 250 μl signal reagent mixture for 2 min, followed by transfer to the Randox Evidence Investigator.

QP biochip image analysis provided background-corrected RLU values. Data corresponding to tracer-only positions (RLU_max_) were used to calculate the % RLU/RLU_max_ values. For QP69 measurements, the data of the two parallel RLU_max_ measurements data were first averaged and then the RLU values of the samples were divided by the resulting RLU_max_ average and expressed as the RLU/RLU_max_ %. For the QP300 kits, only one tracer and two real samples could be tested in one run. Thus, the RLU values of the samples were divided by the RLU_max_ value of the tracer-only sample to provide the RLU/RLU_max_ % parameter for each mAb.

### Statistical Analysis

#### QP69 and QP300 Biochip Measurements

Corrected data sets derived from QP69 and QP300 biochip measurements were used for further significance testing and descriptive statistical analyses utilizing various R software packages (plyr, reshape, and ggplot2).

Venn diagram data were calculated from pooled sample measurements for which the same sample was measured two or three times. Averaged RLU/RLU_max_ % values and differences between the control and case pools were calculated. Those mAbs for which the calculated difference was >30% of the corresponding control RLU/RLU_max_ % were selected. The lists of such mAbs were compared and plotted as Venn diagrams for both the QP69 and QP300 libraries (R software packages: grid, VennDiagram, gplots).

Input variables were presorted by either statistical significance or random forest analysis to build logistic regression models. The performance of the resulting models was tested, and the corresponding ROC curves and AUC values were computed. Models were also validated on defined subsets of databases (R software packages: readxl, pROC).

For correlation analysis, the QP69 corrected dataset was split according to sample dilution and only the data subset of 100-fold diluted samples was used. QP300 measurements derived from corrected data were also used. First, a pairwise Pearson correlation matrix was computed from the complete observations. Then, these matrices were used as input for the generation of differentially clustered heatmaps using R statistical computing packages (RColorBrewer, gplots, corrplot). Publication quality graphs were designed using the ggplot2 package.

kNN and nonlinear SVM algorithms were performed in R and ROC analyses were performed in SPSS. These analyses were done on QP300 and QP69 databases. For the BSI variables, a maximum value was set up to 120. In the cases of duplicated or triplicated samples, a mean value was calculated. Samples were randomly divided into training (66%) and test set (34%). Ten-fold cross-validation was performed in each model. This cross-validation was repeated 3 times in each run. ROC analysis was done on test sets listing AUC, sensitivity, and specificity. Cluster analysis with the Ward method was made for the best BSI variables. Dendrogram and scatter plots were plotted.

#### QPLC21 Biochip Measurements

QPLC21 biochip data statistical evaluation was prepared by SPSS and by R (package randomForest). Lung cancer plasma protein and QPLC21 variables were filtered out by Random Forest and LSVM analysis, then binary logistic regression models were prepared to predict the tumor. ROC analyses were also made for the predictions to detect the utility of the different forecasts.

Nonlinear SVM algorithms were performed in R and ROC analyses were performed in SPSS. Samples were randomly divided into training (70%) and test set (30%). Ten-fold cross-validation was performed in each model. This cross-validation was repeated 3 times in each run.

A further feature selection was performed. BSI variables were compared by Mann Whitney U test, ROC AUC. The best BSI variables with False Discovery Rate <0.01 were selected for the model.

## Results

### Generation, Characterization of Epitome-Specific mAb-Libraries (PlasmaScan and Quantiplasma), Assay Development, and Analytical Validation of PEP

**Fig. 1:**
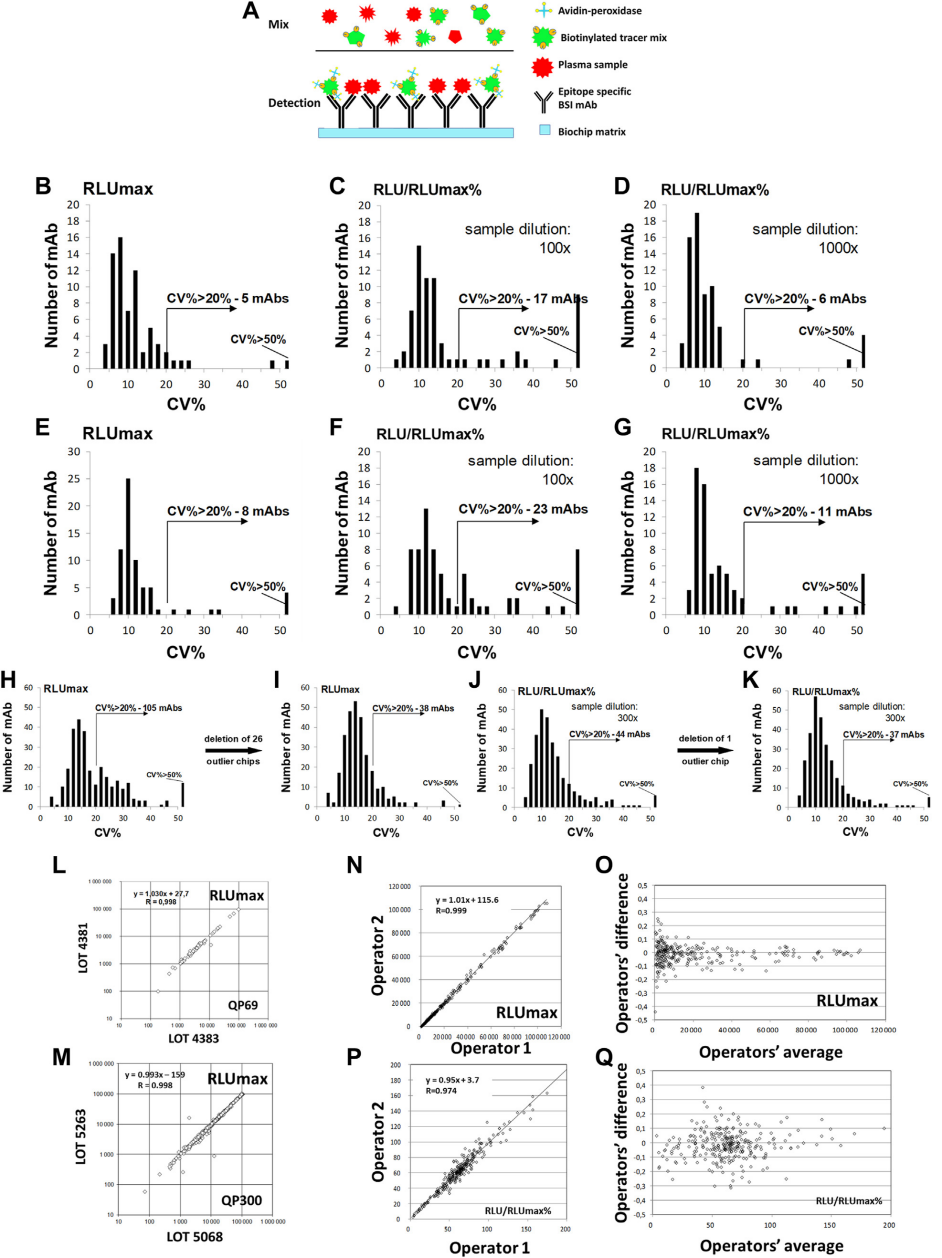
**Analytical validation of the QP69 and QP300 biochips.** Scheme (*A*) shows the principle of the single-binder capture inhibition assay (sbCIA). The reproducibility of the QP69 and QP300 mAb arrays was tested, and the results were plotted as histograms showing the number of antibodies with the given CV (%). Arrows indicate the number of antibodies performing with >20% CV. Intra-assay (*B*–*D*) and inter-assay (*E*–*G*) evaluation of the RLUmax (*B* and *E*) values obtained without plasma competition and the RLU/RLUmax values (%) obtained with competition using 100-fold (*C* and *F*) and 1000-fold (*D* and *G*) diluted plasma on QP69 mAbs spotted onto the Randox biochip array. Inter-assay test results for the RLUmax (*H* and *I*) and RLU/RLUmax (%) (*J* and *K*) values obtained with competition using 300-fold diluted plasma for the QP300 mAbs. Lot-to-lot (*L* and *M*) variability was measured by comparing the averaged RLUmax values of the QP69 (*L*) and QP300 (*M*) mAbs. Inter-operator variability for the QP300 mAbs was also determined based on the measured RLUmax (*N* and *O*) and RLU/RLUmax % (*P* and *Q*) values obtained with competition using 300-fold diluted plasma.

First, we generated a monoclonal antibody library directed against natural epitopes present on abundant and medium-level expressed human plasma proteins, with concentrations ranging from 1.2 ng/ml to 50 mg/ml. We prepared the mAb library using a complex natural immunogen mix prepared from pooled plasma samples from apparently healthy individuals and patients with untreated cancer and chronic inflammatory diseases (supplemental Fig. S1). For the preparation of the immunogens, we used proteome normalization methods (39) following the depletion of the 12 most abundant proteins (31). Standard hybridoma technology (28, 29) produced individual nascent hybridoma supernatants (≈10,000 in total), which were then tested in a high-throughput micro-volume screening (HTS) capture ELISA assay (30, 40, 41, 42) using abundant protein-depleted, biotinylated, normal pooled human plasma for the selection of hybridomas that produced antibodies reacting with natural protein epitopes in the normal human plasma. We previously reported the discovery of cancer-associated plasma proteins, initially by nascent library screening followed by qualification of the candidates by sandwich ELISA (30, 41). Although the nascent hybridoma library showed a large number of initial candidates (30), many were lost during sandwich ELISA development, indicating the importance and uniqueness of the discrete epitopes and suggesting the need for a methodology that profiles individual epitopes *via* single mAb reagents from discovery through qualification, validation, and assay development. To this end, we developed a single-binder capture inhibition assay (sbCIA) (42), based on immobilized epitope-specific mAbs (Fig. 1*A*) and analytically validated the concept of epitome profiling on the Randox Evidence Investigator instrument, a suitable microarray platform (38). In total, 867 mAbs, the PlasmaScan library (PS), providing a positive signal as nascent hybridoma supernatants in sbCIA assays with biotinylated plasma protein preparations were cloned and IgG was purified and characterized. Among them, 380 mAbs, referred to as the QuantiPlasma (QP) library, displaying linear inhibitory characteristics in the sbCIA, were selected. Next, purified mAbs were printed onto ceramic biochip matrices in a 5 × 5 array format, readable in the Randox Evidence Investigator microarray platform (38). Maximal signal intensity (RLU_max_) was obtained with either tracer prepared from non-depleted plasma for 69 mAbs (QP69 biochip for abundant plasma proteins) or with a tracer from depleted plasma for 290 mAbs (QP300 biochip, with 290 qualifying mAbs for medium-abundancy plasma proteins). Biotinylated tracers were prepared under standardized conditions from a pool of 50 plasma samples obtained from blood donors. Individual plasma samples from controls and patients with cancer were tested to determine RLU/RLU_max_ values used as measures of relative epitope abundance. For analytical validation of the QP69 and QP300 epitope profiling tools, 39 QP69 kits (2106 biochips) and 69 QP300 kits (3726 biochips) were manufactured. Analytical testing of the QP69 and QP300 biochips showed intra-assay, interassay, interoperator, and interbatch variability of CV < 20% for 86% and 84% of the mAbs, respectively, following the initial optimization for tracer dilution and data normalization (Fig. 1, *B*–*Q*, Method S1, supplemental Tables S2 and S3). In subsequent experiments, we used analytically qualifying (CV <20%) mAbs exclusively.

**Fig. 2:**
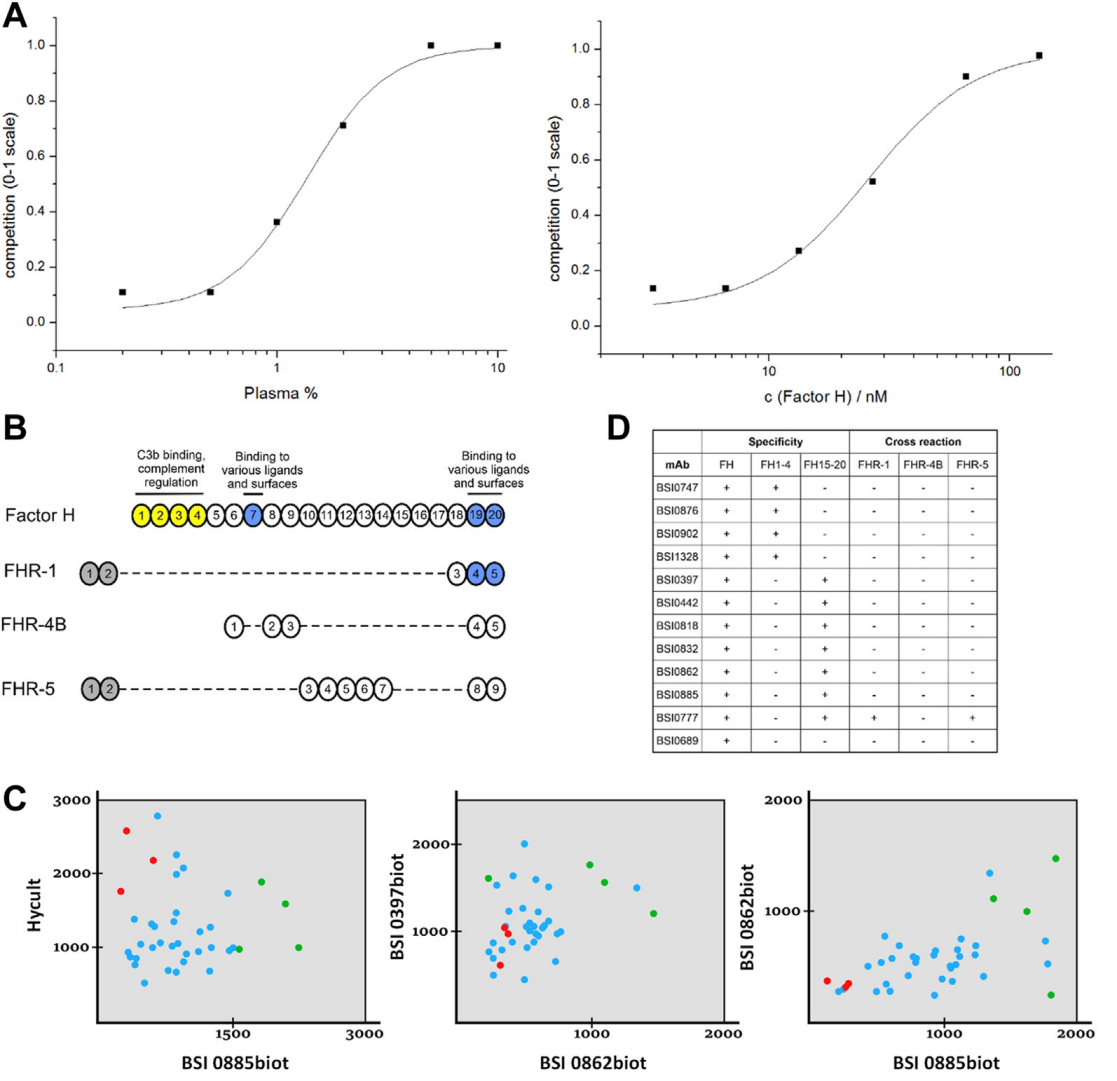
**Mimotope heterogeneity and epitopes of human complement system components.** SPR experiment (*A*) with BSI0442 mAb (factor H) and one of it’s cognate, biotinylated mimotope peptide (#5, immobilized). Binding inhibition curves obtained by competition with pooled human plasma (*left*) or natural CFH purified from plasma (*right*). Domain structure (*B*) of the CFH, CFHL1 and CFHR proteins, the N-terminal domains are shown in *yellow*. *(C)* The CFH concentration (µg/ml) of 37 samples was determined using selected BSI mAbs and a commercial CFH kit (Hycult) in a sandwich ELISA setup. Data for three pairwise comparisons in which seven individual samples were randomly chosen and colored (three *red*, four *green*). Binding test summary (*D*) of QP mAbs that recognize CFH. mAb binding was tested on complete (FH), N-terminal (FH1-4), and C-terminal (FH15–20) fragments of factor H protein and on factor H related proteins (CFHR-1, CFHR-4B, and CFHR-5).

We next assessed the structural redundancy of the cognate antibody binding sites of individual mAbs by randomly choosing 278 mAbs, of which 173 were from the QuantiPlasma library (80% from QP69 and 40% from QP300) for mimotope analysis in standard phage display experiments (33). Mimotopes are epitope-mimicking structures—most frequently peptides—that are able to mimic the reaction between epitopes in antigens and the antibodies they elicit (43). Mimotopes are usually selected from random combinatorial peptide libraries expressed on the surface of bacteriophages. The redundancy of cognate mimotopes was deduced *via* sequence alignments of the mimotope peptides selected from such random peptide libraries. The results indicate minimal mimotope redundancy (15 mAbs with >40% mimotope sequence redundancy out of 173), the maximal value was 92% in a comparison of two mAb pairs, (supplemental Fig. S2). This observation was strengthened by IgG light- and heavy-chain complementarity-determining region sequencing of a few clones (30). The binding affinity of a small set of mAbs was tested with selected mimotope-based synthetic peptides by surface plasmon resonance (SPR). Peptide selection based on inhibition of Ab-peptide binding by natural cognate antigens (Fig. 2*A*). As expected, and corresponding to our published data (33), the K_D_ ranged from 1.65 × 10^−8^ to 9.98 × 10^−8^ M (Table 1.). The proteins recognized by the individual mAbs were identified by subjecting a total of 174 selected mAbs from the QP library to immunoprecipitation and shotgun mass spectrometry analysis, with or without previous SDS-PAGE. Verification was done with immune assays. The list of identified cognate proteins for the QPLC21 selection is shown in supplemental Table S4. Overall, based on the mimotope redundancy tests, the results show that most of the cognate plasma proteins are recognized by multiple mAbs. The estimated average is three mAbs per protein.

**Table 1:** Binding parameters of selected peptide–mAb binding pairs based on SPR data

Peptide	Antibody	K_D_ (M)
179	Bsi0902	1.65E-08
238	Bsi0818	2.11E-08
238	Bsi0832	2.75E-08
36	Bsi0862	3.19E-08
41	Bsi1328	9.98E-08
18	Bsi0893	2.25E-08
274	Bsi0818	2.68E-08
274	Bsi0832	2.84E-08
5	Bsi0442	4.78E-08

The binding parameters K and KD were calculated for each tested antibody concentration using the BIA evaluation stand-alone software package applying the “two state reaction (conformation change)” model for the peptide-antibody interaction. The K (1/M) parameter was the apparent affinity constant and was calculated using the formula: (ka1/kd1)∗(1+ka2/kd2). The average of the four or five values is summarized in the table for each binding pair.

We then further tested the concept of whether the apparent redundancy observed at the cognate protein level is due to immunogenic epitope heterogeneity detected by the QP mAbs. We choose complement factor H (CFH) as an example because we identified 13 mAbs directed against CFH in PS and QP libraries. We found that 11 CFH-specific mAbs map to structurally and functionally independent domains of CFH by testing a series of deletion mutants (Fig. 2*B*). We next investigated whether molecular heterogeneity is detectable *via* CFH-epitope-specific mAbs by testing independent mAb pairs (Fig. 2*C*) and a commercially available polyclonal antibody to detect CFH in human plasma *via* sandwich ELISA assays (44). Through the use of various combinations of antibodies, we detected previously unreported molecular diversity of CFH in human plasma (Fig. 2, *B*–*D*).

### Discovery of Epitome Variable Panels Associated With Cancer

**Fig. 3:**
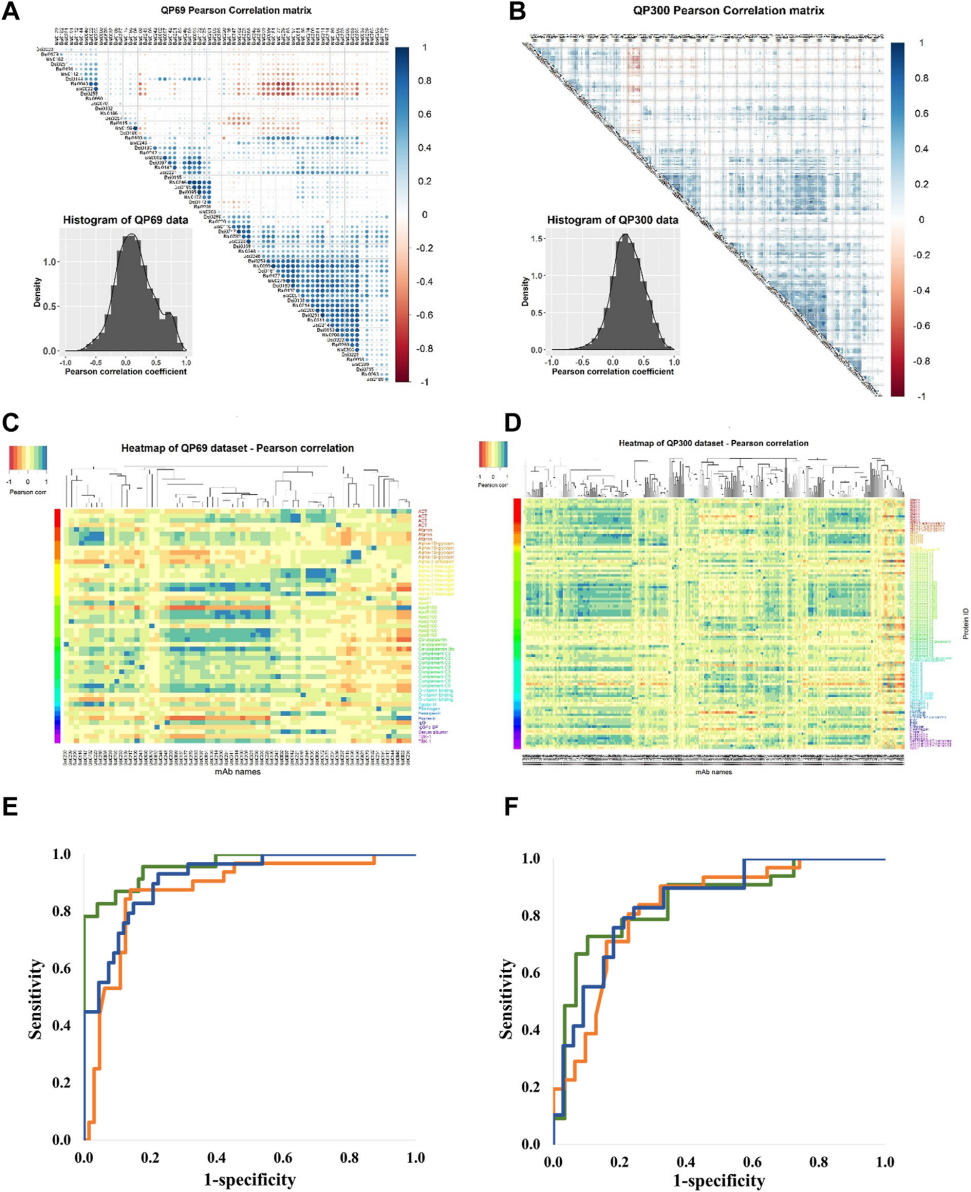
**Epitome profiling of high- and medium-abundance plasma proteins reveals a rich and granular source of cancer biomarkers.** Minimal representational redundancy of epitopes detected by the QP69 and QP300 biochips. Histogram of Pearson correlation coefficients derived from pairwise comparison of the data of individual mAbs/epitopes for QP69 (*A*) and QP300 (*B*). The epitope profiles do not cluster with protein IDs. The computed pairwise Pearson correlation matrix was first subjected to column-wise unsupervised clustering, and the rows were then ordered as a function of the cognate protein ID of the QP69 and QP300 mAb library (*C*: QP69 data set, *D*: QP300 data set using cohorts NKTH and BD). Normalized signal intensity data obtained with LC and control sample sets on QP69 biochip (69 BSI mAb variables) and QP300 (280 BSI mAb variables) along with seven tumor marker data. Samples were randomly divided into training (70%) and test set (30%). Nonlinear SVM models were built on training set incorporating only BSI variables (*blue*), only tumor markers (*brown*) or BSI variables, and tumor markers together (*green*). ROC analyses performed on test sets QP69 (*E*) and QP300 (*F*).

For biomarker discovery, we established a cross-sectional observational biobank cohort of symptomatic, non-treated, lung, breast, and colon cancer patients and apparently healthy, age- and sex-matched individuals to expand the observation to the entire epitome detectable by QP69 and QP300 profiling and to explore the biomarker potential of the epitopes (Table 2). In total, 418 plasma samples, including those of patients with lung, breast, and colon cancer and controls, were profiled. The normalized signal, as the percentage of the RLU/RLU_max_, was determined using the QP69 biochips and 46 lung cancer (LC) samples and an equal number of controls using the QP300 biochips. Epitome-profile datasets (1107 data points for QP69 and 880 for QP300) were first subjected to pairwise correlation analysis of Pearson coefficients with unsupervised clustering (Fig. 3, *A* and *B*). The histograms were symmetrical, with the peak close to “0”, indicating that the individual epitope profiles provide no or only a minimal correlation, with only 6.69% of the Pearson correlation coefficients falling between 0.8 and 1.0. Importantly, the absence of epitome profile clustering by protein IDs was also apparent as supervised clustering of normalized signal (RLU/RLU_max_%) values by protein identifiers did detect classes of similar profiles, but these did not correlate with protein IDs, indicating unexpected epitome granularity (Fig. 3, *C* and *D*). Normalized signal intensity data of LC patients and controls were clustered using the Euclidian distance (linear)–based Ward method (45). Although the cluster analysis revealed specific clusters where control and LC samples were enriched (supplemental Figs. S3–S5), this did not apply to the global dataset. Therefore, we applied SVM models with a non-linear kernel (46), which incorporated all BSI variables in the model and showed good performance ROC AUC 0.895 and 0.916 (Fig. 3, *E* and *F*).

**Table 2:** Details of the lung, breast, and colon cancer and age-matched control sample cohorts

Sample type	Lung cancer		Colon cancer		Breast cancer	
	Control	LC	Control	CC	Control	BC
Sample number	64	69	51	98	51	85
Male	36	41	28	61	0	0
Female	28	28	23	37	51	85
Age (average)	60.9	60.2	60.1	65.8	53.0	58.2
Smoker	31	64	NA	NA	NA	NA
Non smoker	33	4	NA	NA	NA	NA

**Fig. 4:**
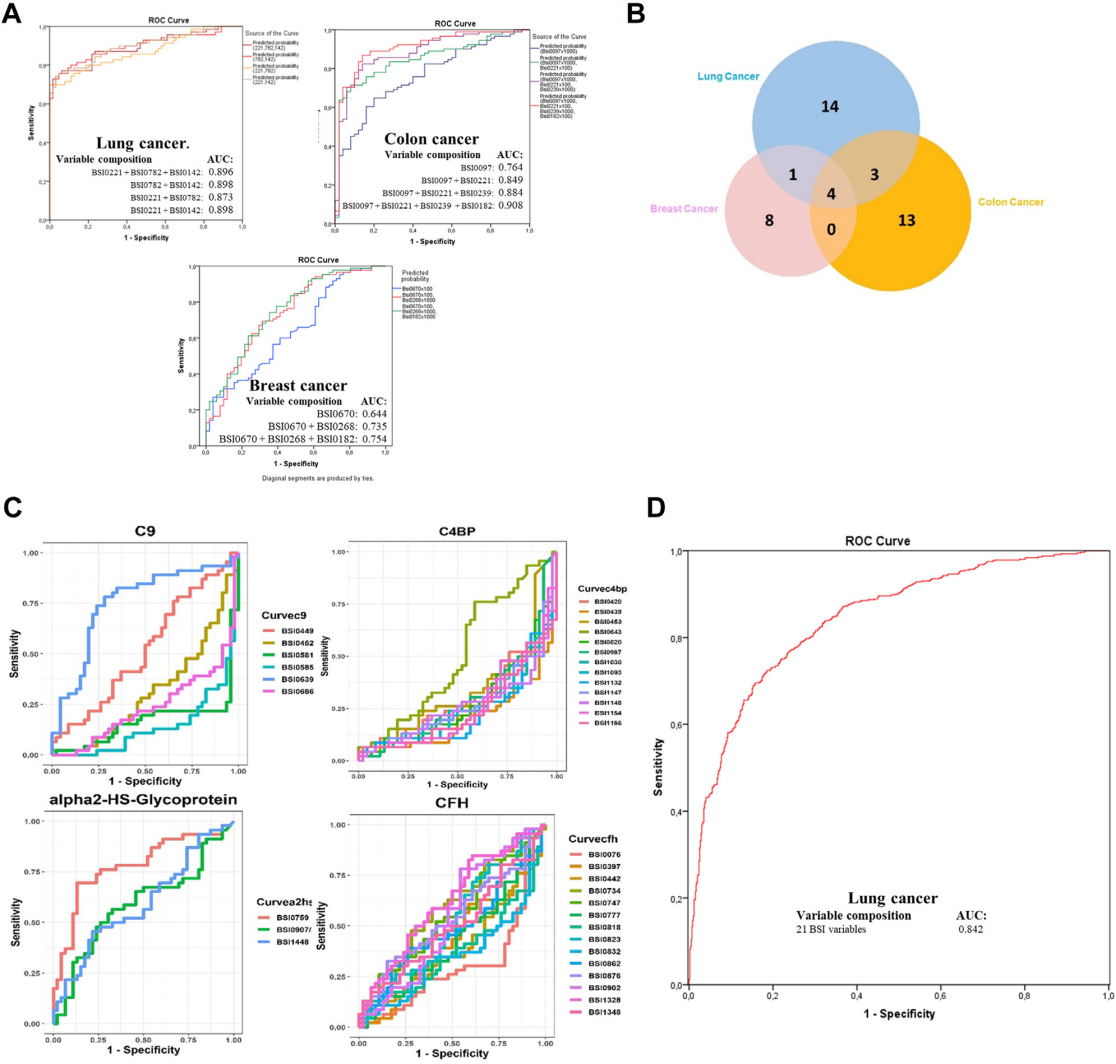
**Plasma proteome epitopes associate with cancer.***A*, Abundant plasma proteins as detected by the QP69 biochip (individual and composite epitopes), showing an association with lung (Bsi0221, Bsi0782, and Bsi0142), colon (Bsi0097, Bsi0221, Bsi0239, and Bsi0182), and breast (Bsi0670, Bsi0268, and Bsi0182) cancers (NKTH cohort). *B*, Cancer-specific epitopes are revealed by the profiling of pooled human plasma samples prepared from samples of lung, breast, and colon cancer patients with both the QP69 and QP300 biochips. Venn diagram shows the number of unique and shared mAbs that discriminate between, in the case of breast (13) and colon (20) cancer, the disease plasma pools *versus* the apparently healthy control sample pool, and in the case of lung cancer, the 22 mAbs selected by epitope profiling data involving lung cancer plasma samples *versus* COPD control samples. *C*, Immunogenic epitopes of a representative set of the LC-associated biomarker proteins C9, C4BP, α2HSGP, and CFH show heterogeneity with respect to association with LC. A fraction of the mAbs that recognize the same protein shows highly positive (*e.g.*, C9-specific mAb Bsi0639) ROC-AUC values, another fraction highly negative (*e.g.*, C9-specific mAb Bsi0686) values, and a third, neutral (*e.g.*, C9-specific mAb Bsi0449) values (please note that in order for the better visualization of disparate, positive, negative or neutral representational changes of specific epitopes, we chose to change the conventional ROC display where absolute values are shown). *D*, LC prediction, the performance of the QPLC21 epitomic panel in predicting LC determined on a 554 LC and 602 control plasma sample of the BD cohort.

To obtain biomarker epitope panels with a reduced number of variables, several statistical methods were used. The analysis of the epitome profiles demonstrated a remarkable association of Bsi0221, Bsi0782, and Bsi0142 individual and composite epitopes with LC, Bsi0097, Bsi0221, Bsi0239, and Bsi0182 with colon cancer, and Bsi0670, Bsi0268, and Bsi0182 with breast cancer (Fig. 4*A*). We tested the specificity of individual epitopes with respect to particular cancer types by testing pooled samples (Table 3) on both the QP69 and the QP300 biochips. The results (Fig. 4*B*) indicate the presence of a higher number of non-shared as opposed to shared epitopes (35 *versus* 8), suggesting the existence of substantial cancer/tissue-type (lung, colon, breast) specificity.

**Table 3:** Properties of the various pools tested in the QP96 and/or QP300 biochip measurements

Code	Cancer type	Subgroup	Number of samples pooled	Gender (male/female)	Age (mean ± SD)
P01.01	Breast	Stage III-IV.	n = 12	0/12	57.5 ± 8.6
P01.02	Breast	Tumor size >3 cm	n = 17	0/17	54.6 ± 14.6
P01.03	Breast	Histologically infiltrative	n = 33	0/31	59.5 ± 11.5
P06.07	Control	Matched for breast cc.	n = 39	0/41	57.4 ± 12
P02.02	Colon	Multiple cancer	n = 19	12/7	65.3 ± 8.5
P02.08	Colon	Stage IIIC-IV.	n = 30	21/9	61.8 ± 12.5
P02.09	Colon	Fecal blood positive	n = 39	24/15	64.7 ± 10.5
P02.17	Colon	Tumor size >6 cm	n = 16	11/5	57.8 ± 13.6
P06.06	Control	Matched for colon cc.	n = 56	30/26	59.1 ± 9.9

We observed that individual LC-associated proteins, such as complement component 9 (C9), C4b-binding protein (C4BP), α2-HS-glycoprotein, and CFH, display both cancer-associated and neutral epitopes. Moreover, with respect to the case of C9 epitopes, we observed both negative, neutral, and positive associations (Fig. 4*C*). Although an association with cancer of each cognate protein has already been reported (47, 48, 49), and furthermore, C9 glycoforms have been suggested to have biomarker value (50, 51), epitopes with characteristic association patterns have not been described.

**Fig. 5:**
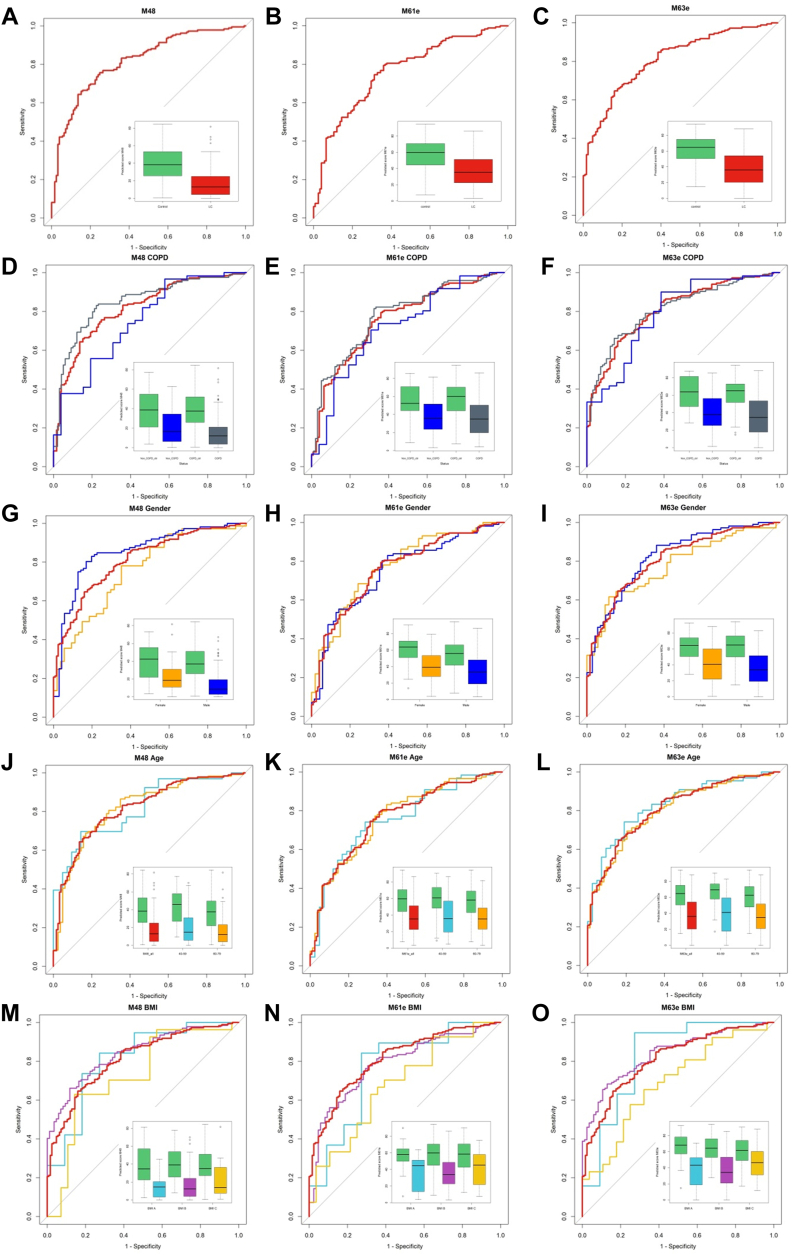
**Impact of confounding factors on the performance of selected mathematical models.** Performance of three selected mathematical models: M48 (*left column*), M61e (*middle column*), and M63e (*right column*) (*A*, *B*, and *C*, color code: LC – *red*, control – *green*). The ROC curve in red shows the entire LC population *versus* all control comparisons in each subfigure. Insets show the spread of the model performance score as boxplots. Subgrouping of the data by COPD (*D*, *E*, and *F*, color code: non COPD – *blue*, COPD – *grey*), gender (*G*, *H*, and *I*, color code: female – *yellow*, male – *blue*), age (*J*, *K*, and *L*, color code: 40–59 years – *light blue*, 60–79 – *yellow*), body mass index (BMI, *M*, *N*, and *O*, color code: BMI-A < 20 kg/m2 – *light blue*, 20 kg/m2 < BMI-B < 30 kg/m2 – purple, 30 kg/m2 < BMI-C – *yellow*).

**Fig. 6:**
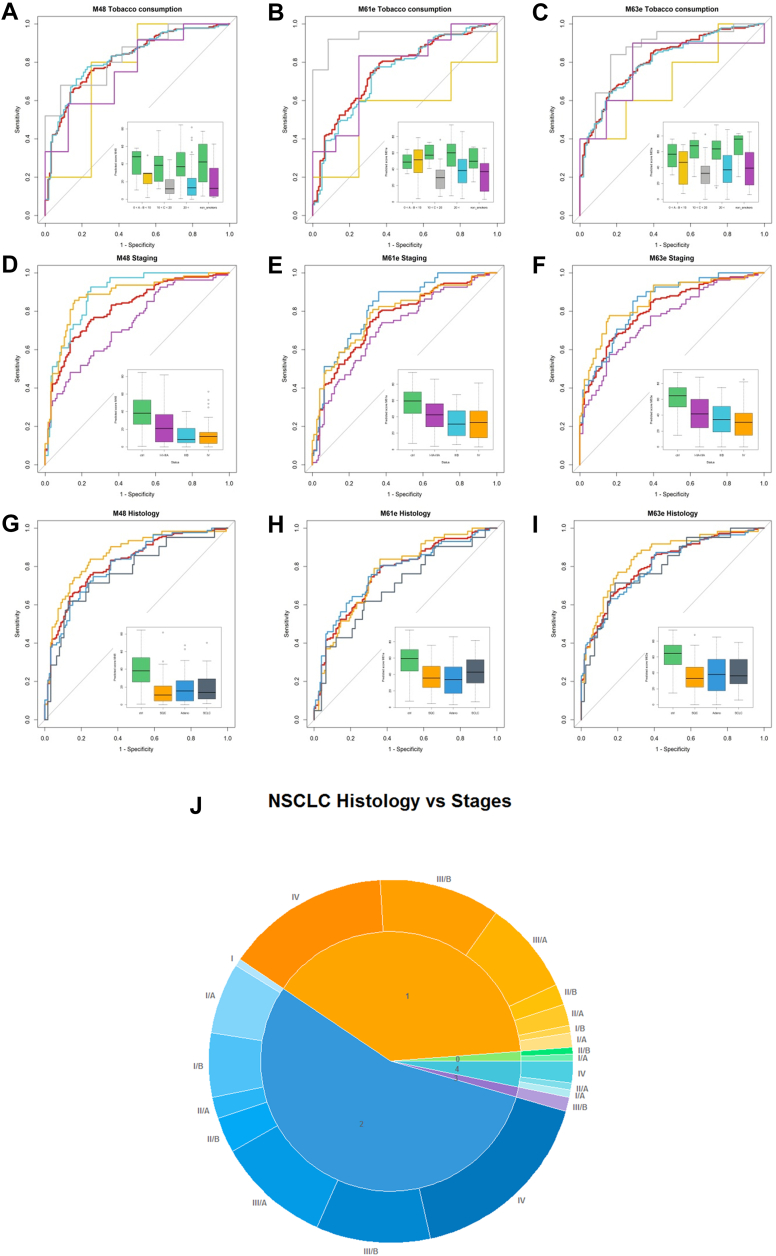
**Impact of confounding factors on the performance of selected mathematical models (continued).** Tobacco consumption in pack-years (PY, *A*, *B*, and *C*, color code: 0–10 PY – *yellow*, 10–20 PY – *grey*, >20 PY – *light blue*, non-smokers – *purple*, LC – *red*, control – *green*), LC stage (*D*, *E*, and *F*, color code: I–IIIA – *purple*, IIIB – *light blue*, IV – *yellow*), and LC histology (*G*, *H*, and *I*, color code: squamous-cell carcinoma (SQC) – *yellow*, adenocarcinoma – *blue*, small-cell carcinoma – *grey*). The pie chart (*J*) shows the fraction of *in situ* squamous cell carcinoma (*light green*), squamous-cell carcinoma (*yellow*), adenocarcinoma (*blue*), adenosquamous cell carcinoma (*purple*), and large cell carcinoma (*light blue*) of non-small-cell LC samples. The outer ring of the chart shows the staging of samples as a hue of the corresponding color (BD cohort).

With the aim of progressing toward the development of a clinically useful blood test with low variable complexity for the detection of LC, we subjected the QP69 and QP300 data to analysis by various machine-learning algorithms (logistic regression, Random Forest, kNN, and linear or non-linear SVM), which delivered epitope panels with varying epitope membership. Based on performance characteristics, we selected 21 epitopes (Table 4, supplemental Table S4) for the production of the LC-specific QuantiPlasma biochip (QPLC21). We then tested 554 plasma samples from symptomatic patients with LC and 602 controls (Table 5) using the QPLC21 biochip. The performance of the QPLC21 biochip validated our previous results obtained with the QP69 and QP300 biochips and provided an accuracy of 0.842 (AUC of ROC analysis, Fig. 4*D*). We next retested the 21 mAbs from the QPLC21 biochip in along with 6 cancer biomarkers (cancer antigen 125 [CA125], carcinoembryonic antigen [CEA], cyfra 21 to 1 [CYFRA], tissue polypeptide antigen [TPAM], C-reactive protein [CRP] and human epididymis protein 4 [HE4]) on a matched sample cohort to reduce the number of variables. Statistical analysis resulted in 72 different functions, composed of different epitopes and clinical variables (supplemental Table S5), in which the detection of clinically operable (Stage I–IIIA) LC was achieved with an accuracy (ROC AUC) ranging from 0.657 to 0.761, while the accuracy for the detection of late stage (IIIB–IV) ranged from 0.759 to 0.914. Epitomic variables significantly improved the performance of cancer biomarkers (alone or in panels), from which group only Cyfra 21-1 was a consistent member. We then selected three models, M48, M61e, and M63e, with 5, 9, and 14 variables, respectively, for analysis of the sensitivity for biological and clinical factors and potential confounding effects. These analyses included sex, age, COPD, BMI, smoking habit, and cancer histology (Figs. 5 and 6, with *p*-values presented in supplemental Table S6). M61e and M63e were stable and not affected by sex, age, COPD, BMI, or smoking habit and both detected non-small-cell lung carcinoma (NSCLC) better than small-cell lung carcinoma (SCLC), whereas detection was less accurate for histologically non-classifiable cancers (Fig. 6, *G*–*I*).

**Table 4:** Statistical model parameters of QPLC21

mAb	B	S.E.	Wald	df	Sig.	Exp(B)
BSI0097	0.069	0.038	3.241	1	0.072	1.071
BSI0116	−0.007	0.015	0.221	1	0.638	0.993
BSI0142	−0.002	0.025	0.008	1	0.928	0.998
BSI0144	0.020	0.029	0.463	1	0.496	1.020
BSI0186	−0.070	0.015	21.517	1	0.000	0.932
BSI0190	−0.002	0.015	0.017	1	0.895	0.998
BSI0221	0.048	0.030	2.563	1	0.109	1.049
BSI0439	0.043	0.027	2.524	1	0.112	1.044
BSI0581	−0.085	0.018	22.142	1	0.000	0.918
BSI0585	−0.035	0.015	5.476	1	0.019	0.966
BSI0639	0.017	0.010	2.808	1	0.094	1.017
BSI0686	0.045	0.016	8.022	1	0.005	1.046
BSI0759	0.042	0.008	25.229	1	0.000	1.043
BSI0789	−0.033	0.014	5.959	1	0.015	0.967
BSI2487	0.053	0.014	13.890	1	0.000	1.054
BSI1154	−0.116	0.032	13.228	1	0.000	0.890
BSI1186	0.040	0.029	1.970	1	0.160	1.041
BSI1517	−0.014	0.009	2.437	1	0.118	0.986
BSI1328	−0.008	0.005	2.601	1	0.107	0.992
BSI0300	0.033	0.013	6.396	1	0.011	1.034
BSI0782	−0.009	0.020	0.200	1	0.654	0.991
Constant	0.754	1.007	0.561	1	0.454	2.126

A logistic regression model was built incorporating all QPLC21 measurement data for the variables of all 21 BSI mAbs. The table shows the following statistical parameters for each variable: B: coefficient of the constant, S.E.: standard error of the coefficient, Wald: Wald chi-square test, df: degrees of freedom, Sig: *p* value. Exp(B): exponentiation of the B coefficient.

**Table 5:** Summary of the 1156 plasma-sample cohort

Grouping factor	Details	Count	Age average
Sample type	Control	602	60.7
	LC	554	62.2
Gender	Female	507	60.2
	Male	649	62.3
Ethnicity	Roma	84	59.6
	Caucasian	802	62.6
	Not available	270	58.6
COPD (GOLD 2012 criteria)	Non COPD	289	61.7
	A	111	62.5
	B	228	61.9
	C	119	63.6
	D	137	62.8
	Not available	272	58.7
Smoking (pack year)	Non smoker	188	61.2
	A	43	57.1
	B	56	60.1
	C	143	58.6
	D	719	62. 4
	Not available	7	57.0
BMI	A	133	59.6
	B	715	61.8
	C	251	61.5
	Not available	57	60.1
Stage	I	3	55.0
	I/A	23	62.6
	I/B	20	62.3
	II	1	54.0
	II/A	17	61.2
	II/B	15	65.8
	III	1	58.0
	III/A	61	60.8
	III/B	112	62.0
	IV	274	62.4
	Not available	28	63.4
Histology code	0	4	65.8
	1	139	62.6
	2	228	61.3
	3	6	66. 7
	4	7	64.7
	5	99	62.2
	6	0	
	7	0	
	8	0	
	9	6	56.2
	10	13	67.8
	Not available	51	63.3

Classification of the COPD sample status was based on 2012 GOLD criteria. Smoking habit groups: 0 < A < 5 pack years, 5 pack years < B < 10 pack years, 10 pack years < C < 20 pack years, 20 pack years < D; # BMI groups: A < 20 kg/m2, 20 kg/m2 < B < 30 kg/m2, 30 kg/m2 < C; & Histology code: 0: squamous cell carcinoma (*in situ*), 1: squamous cell carcinoma, 2: adenocarcinoma, 3: adenosquamous carcinoma, 4: large-cell carcinoma, 5: small-cell lung cancer (SCLC), 6: NSCLC-SCLC combined form, 7: mucoepidermoid cystic carcinoma, 8: adenoid cystic carcinoma, 9: carcinoid, and 10: NSCLC not otherwise specified (NOS).

All ROC curves, cancer specificity, and sensitivity results of the algorithms described refer exclusively to the relevant clinical cohorts used in the experiments. Therefore, the results presented here address and show the goodness of statistical models and are independent of disease prevalence in epidemiologic studies of cancer populations.

## Discussion

Regarding PEP translatability for cancer-associated biomarker discovery, profiling the plasma proteome with QP libraries shows surprisingly high biomarker value at the level of discrete protein epitopes. Furthermore, epitopes of high- and medium-abundance proteins were informative with respect to the detection of cancer, in contrast to the generally accepted notion that medium- and high-abundance plasma proteins have less value as biomarkers than low-abundance proteins (52). Our results also suggest that in addition to the representational level of proteins, dynamic changes in the accessible amount of specific epitopes contribute significantly to specific discriminatory power for each tested cancer type. Supporting the observation as a generalizable notion, with respect to a single protein, LRG1, we have recently described that epitope-specific autoantibody levels are differentially associated with lung cancer (53). Moreover, authors of the Proteome Atlas described that a specific epitope of histidine-rich glycoprotein recognized by antibody Bsi0137 (from the PlasmaScan library) is associated with metabolic and genetic markers differently than another epitope (54). Similarly, it has been established that the CD20 epitope, recognized by the mAb (FMC7), but no other epitopes are sensitive to membrane cholesterol which may mask it and that the unmasked FMC7 epitope is specific for a B cell lymphoma subtype (55). With respect to complement system-specific epitopes, we compared complement activity (data not shown, manuscript in preparation) modulatory and biomarker value of specific mAbs and found minimal overlap. A deeper investigation of the molecular mechanisms responsible for epitome variability in human plasma may have important biological implications for understanding the plasma proteome in cancer.

Protein epitope profiling combines hypothesis-free affinity proteomics technology with mass spectrometry and biological validation. The hypothesis-free and extendable process translates efficiently as it delivers biomarker candidates (shown in the manuscript) and therapeutics candidates (manuscript in preparation).

Monitoring epitope dynamics using QP mAb library technology may contribute to the development of multivariate epitomic panel-based biochips for various standalone or complementary diagnostic applications, including the early detection of LC.

In conclusion, current proteomic profiling technologies fail to detect epitope variability beyond the detection of individual proteins (13). Here, with PEP of the human plasma, we establish that in contrast to popular proteomic technologies, PEP detects clinically valuable heterogeneity and biologically relevant epitopes.

## Data Availability

LC-MS/MS data available at: https://massive.ucsd.edu, Dataset Identifier: MSV000091602.

Further data availability requests should be addressed to Laszlo Takacs and Jozsef Lazar.

## Supplemental data

This article contains supplemental data (32).

## Conflict of interest

The authors declare no conflict of interest.
