# Supplementary material for: Large-Scale Plasma Proteome Epitome Profiling is an Efficient Tool for the Discovery of Cancer Biomarkers
Source: Mol Cell Proteomics. 2023 May 20;22(7):100580. doi: 10.1016/j.mcpro.2023.100580 (PMC10319867; doi:10.1016/j.mcpro.2023.100580)
Supplement: Supplemental Data [file mmc1.pdf]

Large scale plasma proteome epitome profiling is an efficient tool for the discovery of cancer biomarkers

Authors:

Jozsef Lazar, Peter Antal-Szalmas, Istvan Kurucz, Annamaria Ferenczi, Mihaly Jozsi, Ilona Tornyi, Monika Muller, Janos Fekete, John Lamont, Peter FitzGerald, Anna Gall-Debreceni, Janos Kadas, Andras Vida, Nadege Tardieu, Yann Kieffer, Anne Jullien, Mariana Guergova-Kuras, William Hempel, Andras Kovacs, Tamas Kardos, Nora Bittner, Eszter Csanky[0], Maria Szilasi, Gyorgy Losonczy, Klara Szondy, Gabriella Galffy, Edit Csada, Klara Szalontai, Attila Somfay, David Malka, Paul Cottu, Krisztina Bogos and Laszlo Takacs

List of Supplementary Data

1. Supplementary Methods 1 - Analytical evaluation of the QP69 and QP300 arrays
2. Figure S1. - Immunogen sources for hybridoma production.
3. Figure S2. - Redundancy distribution of BSI mAbs
4. Figure S3. - Cluster analysis of QP69 dataset
5. Figure S4. - Cluster analysis of QP300 dataset
6. Figure S5. - Cluster-4 of QP300 dataset dendrogram
7. Table S1. - Properties of QP69 and QP300 BSI mAbs.
8. Table S2. - Summary of the QP69 mAb analytical evaluation

9. Table S3. - Summary of the QP300 mAb analytical evaluation
10. Table S4. - Properties of QPLC21 BSI mAbs
11. Table S5. - Summary table of the ROC curve analysis of the retesting of QPLC21 for all tested statistical models
12. Table S6. - Summary table of the ROC curve analysis using the M48, M61e and M63e statistical models

Supplementary Methods

Supplementary Methods 1

Analytical evaluation of the QP69 and QP300 arrays

Intra-assay evaluation of the RLUmax values in the QP69 array

In one experiment, the biotinylated plasma tracer was incubated in the absence of a competing plasma sample ("tracer only" sample) in ten individual triplet-chips of the QP69 kit. The mean, SD and CV% of the 10 RLUmax values were calculated for each mAb. The CV% was below 20% in the case of 64/69 (92.8%) mAbs, while 5 mAbs provided CV%>20% where one outlier of the ten measured values distorted the analysis; the omission of these outliers reduced the CV% to or below 10%. (Figure 1. B, Supplementary Table 2).

Inter-assay evaluation of the RLUmax values in the QP69 array

In 25 QP69 experiments, we compared plasma samples of patients with cancer and healthy controls. In every experiment, the "tracer only" sample was measured in duplicate,

and an average was calculated from the 2 RLUmax intensities. The mean, SD and CV% of these 25 RLUmax daily average values were calculated for each mAb. The CV% was below 20% in the case of 61/69 (88.4%) mAbs. The omission of one outlier daily average value from 25 reduced the CV% to or below 20% in the case of 4 problematic mAbs (Figure 1. E, Supplementary Table 2).

Intra-assay evaluation of the RLU/RLUmax% values of one pooled control sample in the QP69 array

Two different dilutions (100x and 1000x) of a pooled control plasma sample (quality control - QC - sample) were tested 8times in one QP69 assay. The RLU/RLUmax% values were determined for each mAb and the mean, SD and CV% of these 8 RLU to RLUmax rates were calculated.

The intra-assay CV% of RLU/RLUmax% – using 100x dilution of the QC sample – was above 20% in the case of 17/69 (24.6%) mAbs. From these mAbs, in 3 cases 1 outlier of the eight measured values distorted the analysis; the omission of these outliers reduced the CV% below 20%. From the remaining 14 problematic mAbs, in 7 cases the measured RLU/RLUmax% value was very low (<10%), indicating that the concentration of the measured analyte is too high in the plasma, and a higher plasma dilution is required to achieve reproducible result (Figure 1. C, Supplementary Table 2).

The intra-assay CV% of RLU/RLUmax% values using 1000x dilution of the QC sample showed better results. The CV% was above 20% in the case of 6/69 mAbs (8.7%). From these mAbs, in 4 cases 1 outlier of the eight measured values distorted the analysis;

the omission of these outliers reduced the CV% to and below 20%. (Figure 1. D, Supplementary Table 2).

Inter-assay evaluation of the RLU/RLUmax% values of one pooled control sample in the QP69 array

Two different dilutions (100x and 1000x) of a pooled control plasma (QC) sample were tested in ten independent QP69 assays in 10 different days. The mean, SD and CV% of the 10 RLU/RLUmax% values were calculated for each mAb.

The inter-assay CV% of RLU/RLUmax% – using 100x dilution of the sample – was above 20% in the case 23/69 (33.3%) mAbs. From them, in the case of 12 mAbs the omission of 1 outlier reduced the CV% to or below 20%. From the other 11 mAbs (with CV%>20%), the measured RLU/RLUmax% value was very low (<10%) in the case of 4 mAbs, indicating that the concentration of the measured analyte is too high in the plasma, and a higher dilution is required to achieve reproducible result (Figure 1. F, Supplementary Table 2).

The inter-assay CV% of RLU/RLUmax values using 1000x dilution of the QC sample showed better results. The CV% was above 20% in the case of 11 mAbs, from which in 5 cases 1 outlier of the ten measured values distorted the analysis. (Figure 1. G, Supplementary Table 2).

Lot-to-lot variation of the RLUmax values in the QP69 array

In order to evaluate the lot-to-lot reproducibility of the QP69 kit, the mean RLUmax values measured with lot 4383 (25 experimental days) and lot 4381 (14 experimental days) were calculated for each mAb. Lots 4383 and 4381 were produced at the same time in January of 2012. The mean RLUmax values of lots 4383 and 4381 were very much similar (R=0.998), except one mAb (Bsi0177) (Figure 1. L).

Analytical evaluation of the QP69 kit – summary

The overall reproducibility of data provided by the QP69 kit was evaluated mAb-by-mAb based on the intra- and inter-assay CV% results presented above. There are 49 mAbs coated on the chip that produced analytically acceptable results in both plasma dilutions (100x, 1000x). In these cases, the intra- and inter-assay CV%-s were higher than 20% only in (maximum) one type of analysis (RLUmax or 100x sample dilution or 1000x sample dilution). In the case of 10 mAbs, the intra- or inter-assay CV%-s were above 20% while testing the 100x dilution of the plasma sample, but below 20% in the presence of the 1000x diluted plasma. In all of these cases, the RLU/RLUmax% values were <10% using the 100x dilution of the plasma sample, indicating that these mAbs can be tested only with 1000x diluted plasma (Bsi0144, Bsi0002, Bsi0095, Bsi0097, Bsi0172, Bsi0142, Bsi0173, Bsi0195, Bsi0246, Bsi0257). Finally, 10 mAbs provided very high variability in most of the tests performed; therefore, the results presented by them analytically are not reliable (Bsi0032, Bsi0765, Bsi0179, Bsi0289, Bsi0263, Bsi0239, Bsi0203, Bsi0243, Bsi0186, Bsi0177) (Supplementary Table 2).

Inter-assay evaluation of the RLUmax values in the QP300 array

In 56 QP300 experiments, we compared plasma samples of patients with cancer and healthy controls. In every experiment, the “tracer only” sample was measured once and the mean, SD and CV% of these 56 daily RLUmax values were calculated for each mAb. The CV% was above 20% in the case of 105/290 (36.2%) mAbs. Critically evaluating the data, we realized that certain chips in certain experiments randomly provided large number of outliers compared to their 56-day-average. Altogether 26 such chips (from the 1,008 tested) were identified where more than one third of the plotted mAbs generated an RLUmax signal that was more than 50% higher or lower than the corresponding 56-daily-average RLUmax. Omitting all of the data of these 26 chips, the reproducibility became much better. The CV% was above 20% only in the case of 38/290 (13.1%) mAbs. From these 38 mAbs an RLUmax signal lower than 500 was measured in the case of 5 mAbs. (Figure 1. H, i, Supplementary Table 3).

Inter-assay evaluation of the RLU/RLUmax% values in the QP300 array

One dilution (300x) of a pooled quality control (QC) plasma sample was tested in ten independent QP300 assays in 10 different experiments. The mean, SD and CV% of the 10 RLU/RLUmax% values were calculated for each mAb.

The CV% was above 20% in the case of 44/290 (15.1%) mAbs. Critically evaluating the data, we found only 1 chip (from the 540 tested) where more than one third of the plotted mAbs generated an RLU/RLUmax% value that was more than 50% higher or lower than the corresponding 10-daily-average RLU/RLUmax%. Omitting all of the data of this chip the CV% was above 20% in the case of only 37/290 (12.8%) mAbs. From these mAbs, in 15 cases one outlier of the ten measured values distorted the analysis; the

omission of these outliers reduced the CV% below 20%. (Figure 1. J, K, Supplementary Table 3).

Inter-operator variability of the RLUmax values in the QP300 array

The 56 QP300 experiments were managed by two technicians, utilizing equal number of kits (28-28 each). The average of the 28 RLUmax values was calculated for each mAb in the case of both operators. Correlation and Bland-Altman analyses was performed to compare the performance of the two technicians. The coefficient (R=0.999) and the slope of the correlation curve (a=1.01) suggested very similar results, furthermore the Bland-Altman plot showed >20% difference between the 2 operators only in the case of 7/290 mAbs (Bsi1641, Bsi0218/2, Bsi0348, Bsi2042, Bsi1084, Bsi0919, Bsi0745). (Figure 1. N, O, Supplementary Table 3).

Inter-operator variability of the RLU/RLUmax% values in the QP300 array

One dilution (300x) of a pooled quality control (QC) plasma sample was tested in ten independent QP300 assays in 10 different experiments, performed by two technicians in an equal rate. The average of the 5 RLU/RLUmax% values was calculated for each mAb in the case of both operators. Correlation and Bland-Altman analyses was performed to compare the performance of the two technicians. The RLU/RLUmax% values differed largely in the case of 5 mAbs (Bsi0571, Bsi1815, Bsi0745, Bsi1475, Bsi0846), therefore, these outlier values were omitted from the analysis. The coefficient (R=0.973) and the

slope of the correlation curve (a=0.95) suggested reasonably similar results, furthermore the Bland-Altman plot showed >20% difference between the 2 technicians only in the case of 15/285 mAbs (Bsi0786, Bsi0749, Bsi0314, Bsi0709, Bsi1779, Bsi0910, Bsi1071, Bsi1680, Bsi0731, Bsi0818, Bsi1034, Bsi0654, Bsi0639, Bsi0501, Bsi1060). (Figure 1. P, Q).

Lot-to-lot variation of the RLUmax values in the QP300 array

In order to evaluate the lot-to-lot reproducibility of the QP300 kit the mean RLUmax values measured with lot 5068 (54 experimental days) and lot 5263 (13 experimental days) were calculated for each mAb. These lots were produced at the same time in October of 2013. The mean RLUmax values were very much similar (R=0.998) (Figure 1. M). Only 3 mAbs (Bsi0778, Bsi1680, Bsi1814) out of 290 showed marked lot-to-lot variation.

Analytical evaluation of the QP300 kit – summary

The overall reproducibility of data provided by the QP300 kit was evaluated mAb-by-mAb based on the inter-assay CV%, lot-to-lot and inter-operator variability of RLU/RLUmax% and RLUmax results presented above. Since random errors of 1 chip in the case of RLU to RLUmax rate and 26 chips in the case of the RLUmax testing distorted the analysis, the results of these chips were omitted. If in the case of a certain mAb the corrected inter-assay CV% was below 20% for both RLUmax and RLU/RLUmax% - in the presence of maximum 1 outlier value -, the performance of this mAb was rated as “good”. If the CV% was above 20% for any of the two parameters after correction, the

mAb was named as “not good”. Based on this evaluation, 243 mAbs provided good reproducibility, while 47 showed high analytical variability (Supplementary Table 3).

Evaluation of pooled patient and control samples by the QP69 and QP300 arrays

To test specificity of individual epitopes with respect to particular cancer types, pooled samples were tested on both QP69 and QP300 biochips. Sample pools were prepared by mixing individual samples preferably with progressive - large, infiltrative - cancer. The properties of the tested pools are listed in Table 4.

Evaluation of pools with the QP69 kit[1]

In each cancer type 3-3 pools and a proper control pool was evaluated using the QP69 array. Each pool was measured once on three independent days. First the RLUmax values of these measurements were evaluated. Outlier cases were identified when the actual RLUmax of a mAb showed higher than 50% deviation from the average calculated from all of the experiments. These RLUmax and the corresponding RLU/RLUmax% values (115/6624) were omitted from the analysis. Then mean and CV% was calculated from the 3 individual measurements for each mAb and pool and if the variation of them was higher than CV%>20%, an outlier of the 3 values were identified and omitted. The outlier was defined as a higher value than the mean ± 3SD of the other two determinations. In this way the omitted number of data was 318/6624, showing aggregation in a group of 27/138 mAbs.

[1] Please note, that in addition to results presented in the manuscript, the numbers shown here include experiments using samples from ovarian and prostate sample pools, which we do not show, because the low number of individual samples used for pooling.

Finally, 2208 average RLU/RLUmax% values - calculated from 6191 individual data - of the 12 cancer and 4 matched control sample pools were compared by mAb using t-test or the Mann-Whitney U test. mAbs showing significantly different values between each 3 pools of a certain cancer and the proper control pool were selected as effective in the case of a given cancer. The effective markers were the following: colon cancer - Bsi0180, Bsi0182, Bsi0183; breast cancer - Bsi0281, Bsi0660. Cancer specificity of the effective mAbs is presented as a Venn diagram on Figure 4. B.

Evaluation of pools with the QP300 kit[1]

In 23 QP300 kits, 1,062 chips were used for the evaluation of 8 control and 10 cancer pools. In the case of breast, ovary and prostate cancer 2-2, in the case of colon cancer 4 sample pools with their properly matched control pools were evaluated twice in separate experiments. In every experiment, the "tracer only" sample was measured once and the mean of these 23 daily RLUmax values were calculated for each mAb. Critically evaluating the data, we realized that certain chips in certain experiments randomly provided large number of RLUmax outliers compared to their 23-day-average. Altogether 16 such chips (from the 414 used for RLUmax measurements) were identified where more than one third of the plotted mAbs generated an RLUmax signal that was more than 50% higher or lower than the corresponding 23-daily-average RLUmax. All of the RLUmax data of these 16 chips (252/6,670), and the corresponding RLU/RLUmax% values (392/10,440) were omitted from the analysis. Then mean was calculated from the 2 individual measurements for each mAb and pool. In 251/4828 cases the 2 single RLU/RLUmax% values showed larger variance (>20% CV) aggregating in a group of 67/290 mAbs, but none of the valuable mAbs was among them. If one RLU/RLUmax% value of the duplicate measurements was omitted from the analysis - because of the rejection of the appropriate RLUmax -, the remaining single determination was used for the statistical analysis.

Finally, 5204 average (or single) RLU/RLUmax% values - calculated from 10,048 individual data - of the 10 cancer and 8 matched control sample pools were compared by mAb using t-test or the Mann-Whitney U Wilcoxon-test. mAbs showing significantly different values between each 2 or 4 pools of a certain cancer and the proper control pool were selected as effective in the case of a given cancer. The effective markers were the following: colon cancer - Bsi0777, Bsi0832, Bsi0738, Bsi1456, Bsi0121, Bsi0652, Bsi1033, Bsi1000; breast cancer - Bsi1821, Bsi1447, Bsi0895, Bsi1085, Bsi0507, Bsi0545, Bsi0548, Bsi0549, Bsi0551, Bsi0624, Bsi0630. Cancer specificity of the effective mAbs is presented as a Venn diagram on Figure 4. B.

Supplementary Figures

Figure S1.

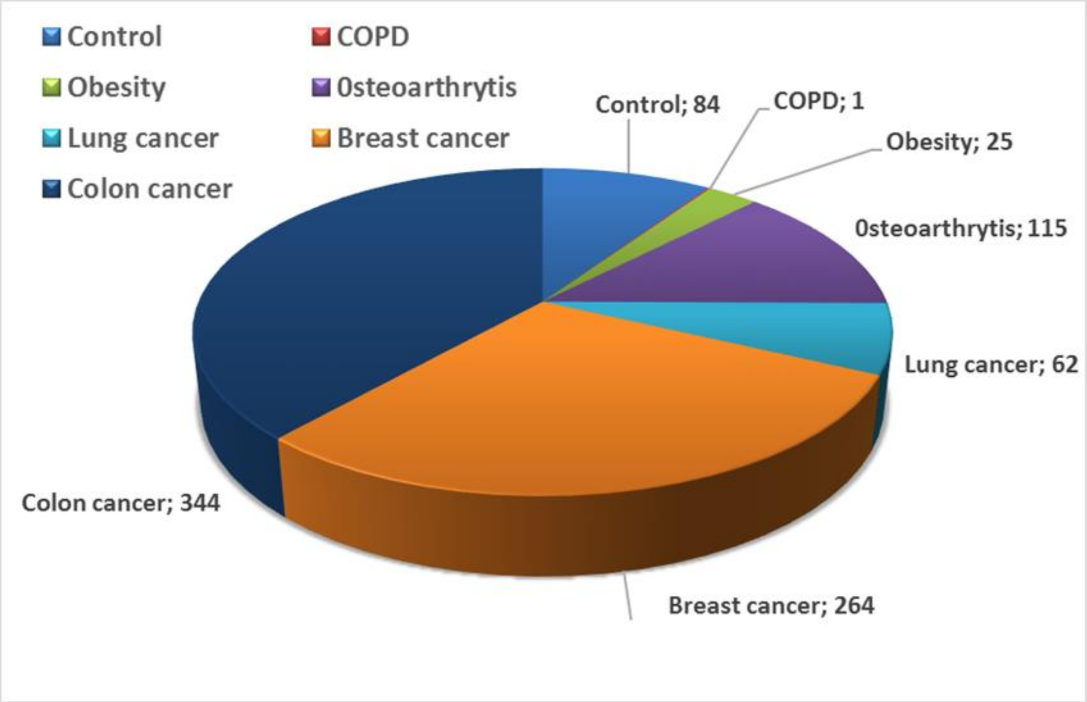

Immunogen sources for hybridoma production. Pie chart showing the number of hybridoma cell lines selected from fusions of SP2/0 BALB/c myeloma cells with splenocytes and lymph-node cells of BALB/c mice immunized with the indicated antigen mixture, as previously described (28).

Figure S2.

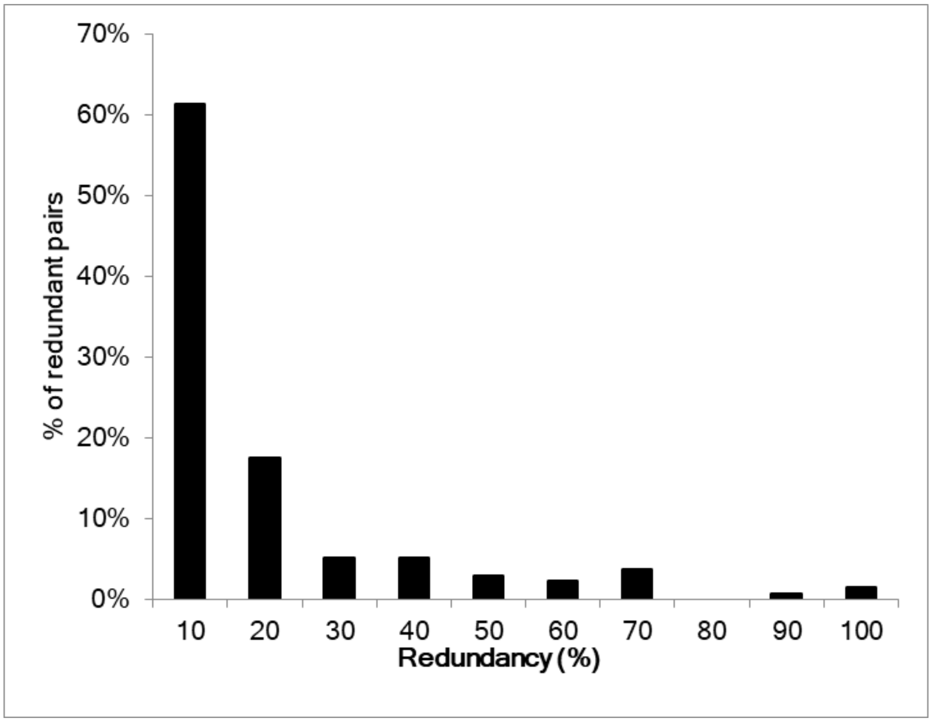

Redundancy distribution of BSI mAbs. Mimotope peptide redundancy distribution of 137 mAb pairs. Redundancy was computed by pairwise comparison of each mAbs' unique mimotope sequences. Column represents the percent of redundant mAb pairs fall into the actual redundancy % bin. Please note, that 10 or 20% relative redundancy means maximum 1 to 4 mimotope peptide identity in the pairwise comparison.

Figure S3.

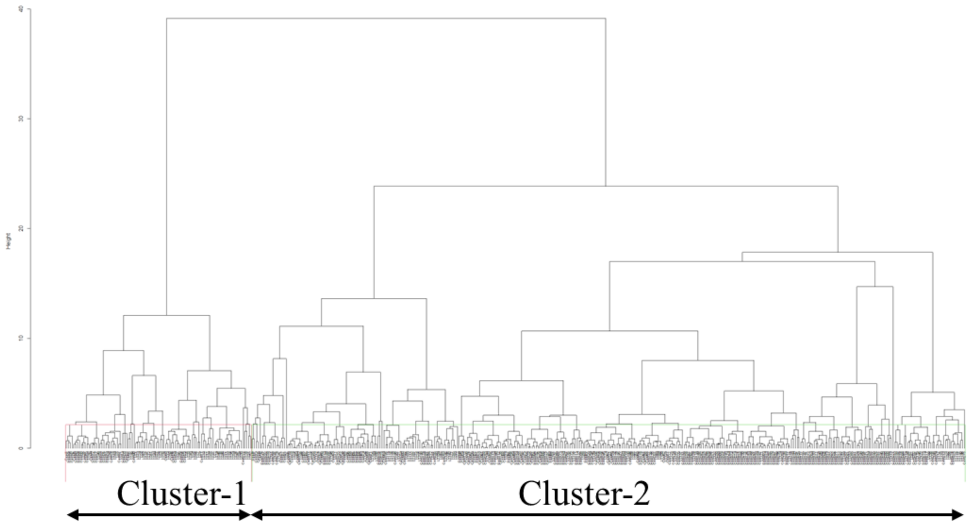

QP 69 top 5 CA cluster		
**Cluster**	**Control**	**LC**
Cluster-1	36.4%	63.6%
Cluster-2	76.3%	23.7%

Cluster analysis of QP69 dataset. Dendrogram shows the result of cluster analysis with Ward method was made for the five best BSI variable data of QP69 dataset. Table summarizes the distribution of sample types as % of all sample in the two subclusters.

Figure S4.

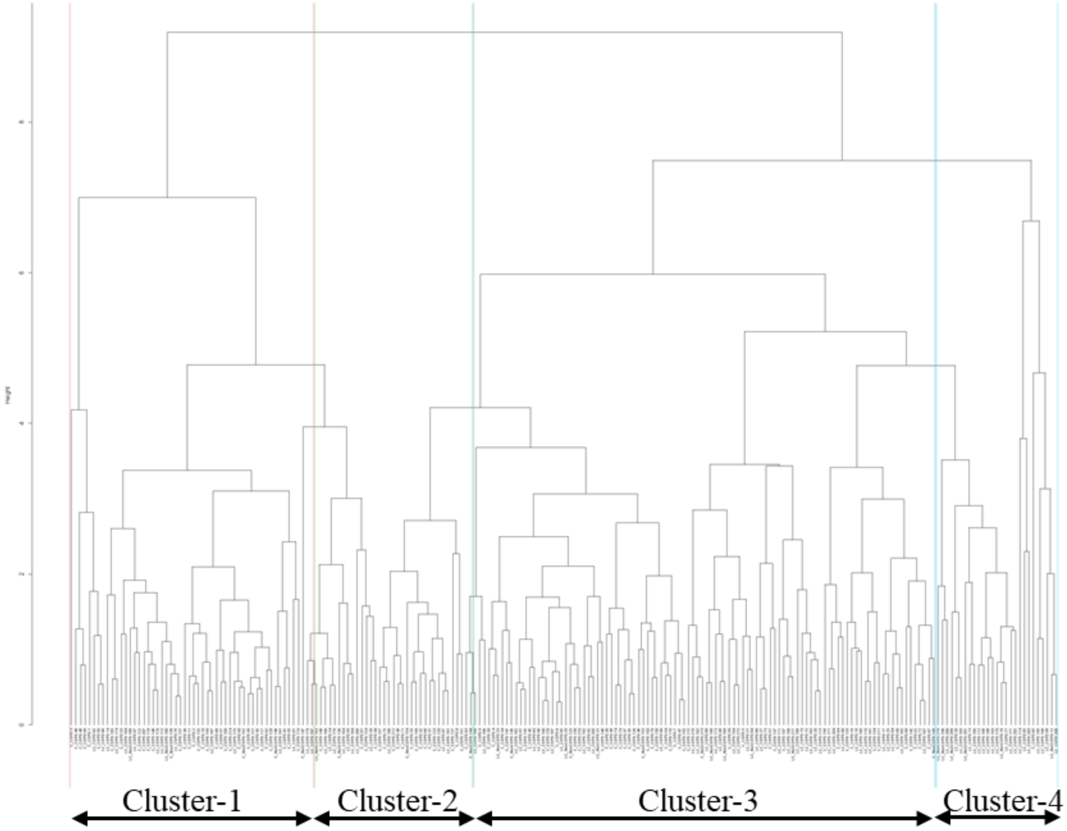

QP 300 top 5 CA cluster				
**Cluster**	**Control COPD**	**Control nonCOPD**	**LC COPD**	**LC nonCOPD**
Cluster-1	61.1%	7.4%	27.8%	3.7%
Cluster-2	57.1%	8.6%	31.4%	2.9%
Cluster-3	46.1%	4.9%	42.2%	6.9%
Cluster-4	3.7%	0.0%	74.1%	22.2%

Cluster analysis of QP300 dataset. Dendrogram shows the result of cluster analysis with Ward method was made for the five best BSI variable data of QP300 dataset. Table summarizes the distribution of sample types as % of all sample in the subclusters.

Figure S5.

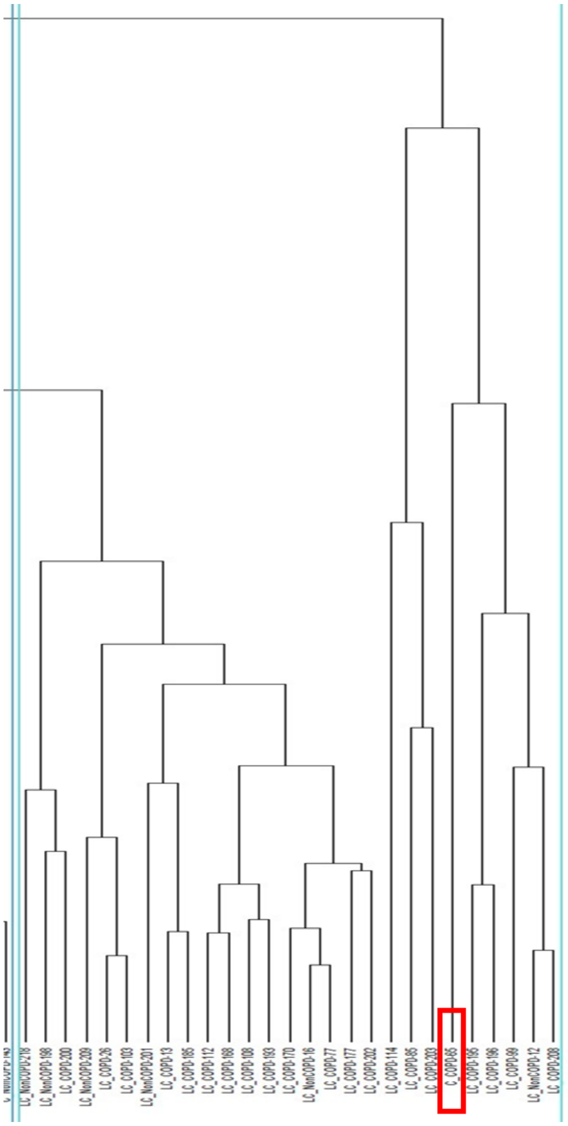

Cluster-4 of QP300 dataset dendrogram. Cluster analysis with Ward method was made for the five best BSI variable data of QP300 dataset. Magnified dendrogram shows the fourth cluster of dendrogram seen on Supplementary Figure 4. Red square indicates the only control sample clustered within QP300 Cluster-4.

Supplementary Tables

Table S1.

Properties of QP69 and QP300 BSI mAbs.

List of QP69 and QP 300 BSI mAbs indicating phage display derived mimotope sequences. mAbs discriminating between breast or colon pools and their control pools and the 22 mAbs of lung cancer and control samples were labelled in the “Cancer discrimination” column. Analytical test results were also indicated.

Table S2.

Summary of the QP69 mAb analytical evaluation.

The overall mAb-by-mAb reproducibility of the data provided by the QP69 kit was evaluated based on the intra- and inter-assay CV% and lot-to-lot analysis results. There were 49 mAbs coated on the chip that produced analytically acceptable results for both plasma dilutions (100x, 1000x). The intra- and inter-assay CV% was > 20% only in maximum one type of analysis (RLU_{max} or RLU/RLU_{max} calculated at the 100x sample dilution or 1000x sample dilution). For 10 mAbs, the intra- or inter-assay CV%s were > 20% when testing the 100x dilution of the plasma sample but < 20% in the presence of the 1000x diluted plasma (except Bsi0257, for which one outlier value resulted in a higher inter-assay CV% for the 1000x dilution). In all such cases, the RLU/RLU_{max} % values were < 10% using the 100x dilution of the plasma sample, indicating that these mAbs can only be tested with 1000x diluted plasma. Finally, ten mAbs yielded very high variability in most of the tests performed; the results obtained with them are, therefore, analytically unreliable.

Table S3.

Summary of the QP300 mAb analytical evaluation.

The overall mAb-by-mAb reproducibility of the data provided by the QP300 kit was evaluated based on the inter-assay CV% of the RLU/RLU_{max} % and RLU_{max} results and on the lot-to-lot and inter-operator analyses. Random errors of one chip for the RLU to RLU_{max} rate and 26 chips for the RLU_{max} testing distorted the analysis. Thus, the results of these chips were omitted. The performance for mAbs for which the corrected inter-assay CV% was < 20% for both the RLU_{max} and RLU/RLU_{max} %, or for which there was a maximum of one outlier value, the omission of which reduced the CV% to < 20%, was rated as “good”. The performance for mAbs for which the CV% was > 20% for any of the two parameters after correction, with more than one outlier value, was rated as “not good”. In case of seven mAbs the lot-to-lot or the inter-operator analyses provided poor results. Based on these evaluations, 243 mAbs provided good reproducibility, whereas 47 showed unsatisfactory analytical variability.

Table S4.

Properties of QPLC21 mAbs.

List of QPLC21 mAbs selected from the pools of QP69 and QP300 BSI mAbs indicating verified cognate protein ID. In the column MS Protein ID - Name those protein names listed which ones met the 5 peptides/protein limit. Protein names matching verified ID indicated with bold fonts. To these hits the following information also included: protein accession number, number of unique peptides assigned to each protein and sequence coverage expressed in % (in case Bsi0186 confidence % is reported) along with the cognate ID replicate measurements and verification methods. Information regarding mAbs also discriminating between breast or colon pools and their control pools were labelled in the "Cancer discrimination" column. Analytical test results and concentration of cognate protein in serum or plasma were also indicated, where any of these had been available.

Table S5.

Summary table of the ROC curve analysis of the retesting of QPLC21 for all tested statistical models.

Seventy-two statistical models (Model #) were built on datasets obtained by sample measurements on QPLC21 biochips with three different tracers (Dataset: QM504 – total tracer, QP507 – depleted tracer, or QM504+QP507 – mixed tracer). Model performance were assessed, as area under the ROC curve on different sample populations (All data, NSCLC data, CE data: controls and early (I. to III/A) stage patients, CL data: controls and late (III.B and IV.) stage patients, NSCLC/CE data: controls and early (I. to III/A) stage patients with NSCLC histology type, NSCLC/CL data controls and late (III.B and IV.) stage patients with NSCLC histology type). AUC values were color scaled from red to green. Model Data (all or indicated subset of sample data), Variable type and Dataset columns defines the data used to build the given model, while the column Selection shows the method by input variable number were decreased. Var_1 - Var_14 are the variables included in the binary logistic model.

Table S6.

Summary table of the ROC curve analysis using the M48, M61e and M63e statistical models.

The significance of models performance on the different sample subpopulations were tested by AUC pairwise comparisons applying the roc.test function (with the following parameters: method = bootstrap and boot.n = 10 000) in the pROC R package, $p < 0.05$ values were highlighted with grey.
